# A novel single nucleotide mutation of *TFL1* alters the plant architecture of *Gossypium arboreum* through changing the pre-mRNA splicing

**DOI:** 10.1007/s00299-023-03086-7

**Published:** 2023-12-29

**Authors:** Ji Liu, Pengfei Miao, Wenqiang Qin, Wei Hu, Zhenzhen Wei, Wusi Ding, Huan Zhang, Zhi Wang

**Affiliations:** 1 National Nanfan Research Institute (Sanya), Chinese Academy of Agricultural Sciences, Sanya, 572024 China; 2 National Key Laboratory of Cotton Bio-breeding and Integrated Utilization, Institute of Cotton Research, Chinese Academy of Agricultural Sciences, Anyang, 455000 China; 3 Hainan Yazhou Bay Seed Laboratory, Sanya, 572025 China

**Keywords:** *Gossypium arboreum*, Determinate growth, *TFL1*, Plant architecture

## Abstract

**Key message:**

A single nucleotide mutation from G to A at the 201st position changed the 5′ splice site and deleted 31 amino acids in the first exon of *GaTFL1*.

**Abstract:**

Growth habit is an important agronomic trait that plays a decisive role in the plant architecture and crop yield. Cotton (*Gossypium*) tends to indeterminate growth, which is unsuitable for the once-over mechanical harvest system. Here, we identified a determinate growth mutant (*dt1*) in *Gossypium arboreum* by EMS mutagenesis, in which the main axis was terminated with the shoot apical meristem (SAM) converted into flowers. The map-based cloning of the *dt1* locus showed a single nucleotide mutation from G to A at the 201st positions in *TERMINAL FLOWER 1* (*GaTFL1*), which changed the alternative RNA 5′ splice site and resulted in 31 amino acids deletion and loss of function of *GaTFL1*. Comparative transcriptomic RNA-Seq analysis identified many transporters responsible for the phytohormones, auxin, sugar, and flavonoids, which may function downstream of *GaTFL1* to involve the plant architecture regulation. These findings indicate a novel alternative splicing mechanism involved in the post-transcriptional modification and *TFL1* may function upstream of the auxin and sugar pathways through mediating their transport to determine the SAM fate and coordinate the vegetative and reproductive development from the SAM of the plant, which provides clues for the *TFL1* mechanism in plant development regulation and provide research strategies for plant architecture improvement.

**Supplementary Information:**

The online version contains supplementary material available at 10.1007/s00299-023-03086-7.

## Introduction

Flowering plants naturally display two types of plant architecture: indeterminate and determinate growth. Considering the indeterminate plants, the main axis can grow unlimitedly and produce flowers on its flank tissues. For determinate plants, the main stem growth is limited and terminated when the shoot apical meristem (SAM) turns into a flower or other reproductive structure and stops continuing to elongate indefinitely (Bradley et al. 1997; Eberwein et al. 2009; Wu et al. 2011).

Determinate growth contributes to a significant and favorable effect on the genetic improvement of many crops such as rapeseed, tomato, cotton, and so on (Jia et al. 2019; Pnueli et al. 1998; Si et al. 2018). The wild type of *Gossypium* spp. has an indeterminate inflorescence and plant architecture. With the realization of cotton production mechanization, the determinate architecture of plants is favorable for cotton mechanical harvest and production, and improving the indeterminate growth habits is a crucial project that breeders and plant scientists encounter. So, revealing the molecular mechanisms underlying the transformation between indeterminate and determinate inflorescence/architecture traits is very important to crop breeding.

The vegetative-reproductive switch is a delicate process mediated by various genetic pathways including photoperiod, gibberellins, nitric oxide, and autonomous pathways (Amasino and Michaels 2010; Boss et al. 2004; Jack 2004). Some key regulators responsible for the floral identity and the fate of SAMs have been identified and studied well. Some homologous phosphatidyl ethanolamine-binding proteins (PEBPs)—TERMINAL FLOWER1 (TFL1)/CENTRORADIALIS (CEN)/SELF-PRUNING (SP) in *Arabidopsis*, *Antirrhinum*, *Cucumber*, tomato as well as *Gossypium barbadense* have been evidenced to play a key role in the maintenance of SAM indeterminate status and floral identity establishment (Bradley et al. 1996, 1997; Shannon and Meeks-Wagner 1991; Wen et al. 2019). It is found that TFL1 and another PEBP-FLOWERING LOCUS T (FT) function antagonistically to regulate the floral transition as mobile RNA or protein in *Arabidopsis*. In the reciprocal pathway, TFL1 represses and FT activates the flowering transition, respectively, consequently playing crucial roles in the inflorescence pattern and plant architecture (Baumann et al. 2015; Shannon and Meeks-Wagner 1991; Wickland and Hanzawa 2015).

In cotton, the homologous of TFL1 and FT have been identified the key roles in plant architecture in *G. hirsutum* and *G. barbadense* (Chen et al. 2018; McGarry et al. 2016; Si et al. 2018). However, the narrow genetic base from repeated selective breeding in allotetraploid cotton restricts the development of novel and high-quality varieties. *Gossypium arboreum* presents some inherent characteristics that the allotetraploid cotton lack, such as the tolerance to drought and salinity and remarkable resistance to pests (e.g., bollworms and leafhoppers) (Nibouche et al. 2008; Tahir et al. 2011) and disease (e.g., rust, fungal and viral) (Akhtar et al. 2010; Wheeler et al. 1999). Moreover, natural *G. arboreum* (also known as Asiatic cotton, a diploid species) exhibits various colorful fibers (e.g., white, green, and tan) and some of the varieties produce high-strength fibers (Mehetre et al. 2003), which provide potential in new cultivar breeding facilitate to the gene pool diversity (Sethi et al. 2015). However, until now, determinate plants or responsible genes has not been reported in *G. arboreum*. Here, we mutated the *G. arboreum* cultivar Shixiya 1 with EMS and screened a determinate mutant—*determinate-growth 1*. The gene cloning with BSA and QTL approaches identified the *DETERMINATE-GROWTH 1*, a novel mutant allele of *TFL1* homolog, reconfirming the key role of *TFL1* in the plant architecture of *Gossypium* spp. including allotetraploid as well as diploid cotton*.* More interestingly, the identified novel mutation site provides clues for a kind of alternative splicing mechanism illumination in the plant.

## Materials and methods

### Plant materials and growth condition

*Gossypium arboreum* acc. Shixiya 1, an indeterminate growth plant, with indeterminate inflorescence and branches, was obtained from South China, Yangtze and Yellow River regions, which was used as a background material to obtained determinate growth mutant *dt1* by EMS mutagenesis. *G. arboreum* acc. MZM971, an indeterminate growth plant, originated in America, which was used as paternal material to construct a F_2_ population. In this study, in view of the fact that Shixiya 1 and MZM971 are unsuitable for gene function verification via the virus-induced gene silencing (VIGS) technology system, *G. arboreum* acc. DQJ, another indeterminate growth plant, planted in Zhao County, Hebei Province, China, was used for the VIGS assay. All the plants were sown in the experimental field of the Institute of Cotton Research, Chinese Academic Agricultural Sciences (ICR, CAAS, Anyang, Henan Province, or Sanya, Hainan Province).

## Identification of the* determinate-growth 1* in EMS-induced mutation population of *G. arboreum*

In order to obtain abundant mutants with novel traits, approximate 15,000 seeds of Shixiya 1 were presoaked in phosphate buffer (100 mM, pH 7.0) for 12 h at 28 ℃. Then, the seeds were soaked in EMS (0.6%, ethyl methane sulfonate) phosphate buffer for 8 h in the dark at 28 ℃ with continuous flip and shake during the period. Subsequently, the seeds were rinsed with distilled water for 30 min with three times to remove residual EMS. Finally, they were sown in the experimental field of ICR, CAAS (Sanya). The M_2_ seeds were harvested by collecting one boll per plant from the surviving 7987 M_1_ plants (53.3% survival). After four generations of self-fertilization, 123 individual lines with visible phenotypes were identified out of 5980 M_7_ plants, and one of which exhibited determinate growth and was named as *determinate-growth 1* (*dt1*).

## F_2_ population construction and super bulked segregant analysis (BSA) sequencing

To clone the causal locus, the *dt1* and MZM 971 were crossed to construct a hybrid line (100 F_1_ lines) in 2016, and an F_2_ population (1000 plants) was generated by self-fertilization of the F_1_ population in 2017. Fresh young leaves were collected from parental lines (MZM 971 and *dt1*), 30 F_2_ individual plants exhibiting indeterminate growth and 30 F_2_ individual plants exhibiting determinate growth. Genomic DNA was extracted from the leaves following the previously reported method (Gong et al. 2018; Li et al. 2019). Two DNA pools were constructed. One pool consisted of DNAs from 30 F_2_ plants showing the dominant phenotype (indeterminate growth), and another pool consisted of DNAs from 30 F_2_ plants showing recessive phenotype (determinate growth). The experimental process is performed according to the standard protocol provided by Illumina for BSA-sequencing. The DNA of each sample was randomly broken into 350 bp fragments by ultrasonic fragmentation to construct the sequencing library. The library was sequenced by Illumina HiSeq after passing the quality inspection. Each sample was sequenced at 30 × coverage of the assembled genome with 150-bp paired-end reads. Clean reads were mapped to the *Gossypium arboreum* (A2) ‘SXY1’ genome CRI-updated_v1 (https://www.cottongen.org/species/Gossypium_arboreum/CRI-A2_genome_v1.0) by BWA after filtering the raw reads. According to the positioning results of clean reads in the reference genome, Picard was used for mark duplicates, GATK for local realignment, base recalibration and other preprocessing to ensure the accuracy of the detected single nucleotide polymorphisms (SNPs). The SNP was further detected by GATK and filtered to obtain the final SNP site set. The average ΔSNP index was calculated based a 1000 kb sliding window with a 10 kb step size to identify regions associated with target trait.

## Development and utilization of KASP marker in the *GaDT1* cloning

According to the distribution and density of SNP physical locations, KASP (Kompetitive Allele Specific PCR) markers design was performed on candidate SNPs using the online platform PolyMarker website (http://www.polymarker.info/) of HuaZhi Biotechnology Co., LTD, and chromosome-specific markers were selected from them. The FAM or HEX fluorescent linker sequence was added to the 5′ end of the KASP markers forward primer, and the FAM linker sequence was 5′-GAAGGTGACCAAGTTCATGCT-3′, the HEX linker sequence was 5′-GAAGGTCGGAGTCAACGGATT-3′. Then, the KASP markers were used for haplotype analysis in the population. If different genotypes of SNPs could be distinguished, the marker was specific and could be used for molecular assisted selection. Based on the BSA result, we found a significant single signal on Chr07 indicating that the determinate growth trait was controlled by a single locus, and designed KASP markers within the candidate region (Chr07: 5.74 Mb to 25.55 Mb).The sequence of primer GH900009 marked on the left side of the positioning interval was: GH900009F_FAM: GAAGGTGACCAAGTTCATGCTGATCATGTTCGTTGCCTTCGTAATG; GH900009F_HEX: GAAGGTCGGAGTCAACGGATTGATCATGTTCGTTGCCTT-CGTAATC; GH900009R: AGGAAGGAGAGATGGACGCTGACAT. The sequence of the primer GH900028 marked on the right was: GH900028F_FAM: GAAGGTGACCAAGTTCATGCTGCAACTAAACGCACCACCCATTAC; GH900028F_HEX: GAAGGTCGGAGTCAACGGATTGCAACTAAACGCACCAC-CCATTAT; GH900028R: TAATATTTCCCTCCCTAGCCCCTCCT.

## RNA-seq and RT‑qPCR analysis

Due to the formation of the terminal flower in the *dt1* mutant, the terminal buds in the different stages T1 (seedling stage, three true leaves), T2 (the early stage of flower bud formation, no visible flower buds) and T3 (flower budding stage, with visible flower buds) were taken from the wild type Shixiya 1 and the mutation line *dt1*, respectively. Total RNA was extracted by NEBNext^®^ Ultra™ Directional RNA Library Prep Kit for Illumina^®^ and used for RNA sequencing. Then two libraries (150 bp-length pair-end reads) were constructed in the same manner and sequenced on Illumina NovaSeq 6000 platform (Novogene, Beijing) following the protocol provided by the company (Illumina, San Diego). Clean reads were aligned to the *G. arboreum* reference genome through the Bowtie software (Li et al. 2009; Langmead and Salzberg 2012). Differentially expressed genes (DEGs) were analyzed by Cufflinks software for FPKM (fragments per kilo bases per million reads) calculation (Trapnell et al. 2010). Benjamini and Hochberg’s approach was used to adjust the *P*-value for the false discovery rate. Genes with an adjusted *P*-value < 0.05 and absolute fold change value > 2 were designated as DEGs. The KAAS (https://www.genome.jp/tools/kaas/) was used for pathway annotation analysis of the DEGs. The RT-qPCR assays were performed by the 2 × Wiz Universal SYBR Green qPCR Master Mix (BCS, No. SPE00005). The *GaHis3* (Cotton_A_11188) was used as the endogenous reference gene for relative quantitation of the gene expression data (Gong et al. 2017). The relative expression levels were calculated using the 2^−ΔΔCt^ method (Livak and Schmittgen 2001). The sequences of each pair of primers used for the different RT-qPCR assays were listed in the Table S1.

## Virus-induced gene silencing (VIGS) assay

For the VIGS assay, we used the TRV vector (i.e., pTRV1 and pTRV2), along with VIGS positive control pTRV2–*PDS*, and negative control (NC) empty vector pTRV2. A 302 bp *GaTFL1* fragment was cloned from the SAM of Shixiya 1 cDNA with the pTRV2-*GaTFL1* F/pTRV2-*GaTFL1* R primer pair (Table S1). The pTRV1 helper plasmid, pTRV2, pTRV2-*PDS*, pTRV2-*GaTFL1* were all introduced into the *Agrobacterium* strain GV3101. VIGS assay and silencing efficiency check were performed with *G. arboretum* DQJ as donor plant according to the previously published protocol (Gong et al. 2017).

## Microscopy observation of SAM in WT and *dt1*

To analyze the SAM of WT and *dt1*, the terminal buds in the different stages S_1_ (1 true leaf), S_2_ (2 true leaves), S_3_ (3 true leaves), S_4_ (4 true leaves) and S_5_ (5 true leaves) were taken from the wild type Shixiya 1 and the mutant line *dt1*, respectively. The samples were fixed in 4% (w/v) paraformaldehyde, 0.25% (w/v) glutaraldehyde and 50 mM sodium phosphate (pH 7.2), dehydrated by passage first through an ethanol series and then through a *t*-butanol series, embedded in paraffin, and finally sectioned to 8 μm by a Leica RM2235 rotary microtome. The paraffin sections were stained with toluidine blue and observed under the Olympus BX53 microscope.

## Results

### The identification of the *determinate-growth 1* mutant

**Fig. 1:**
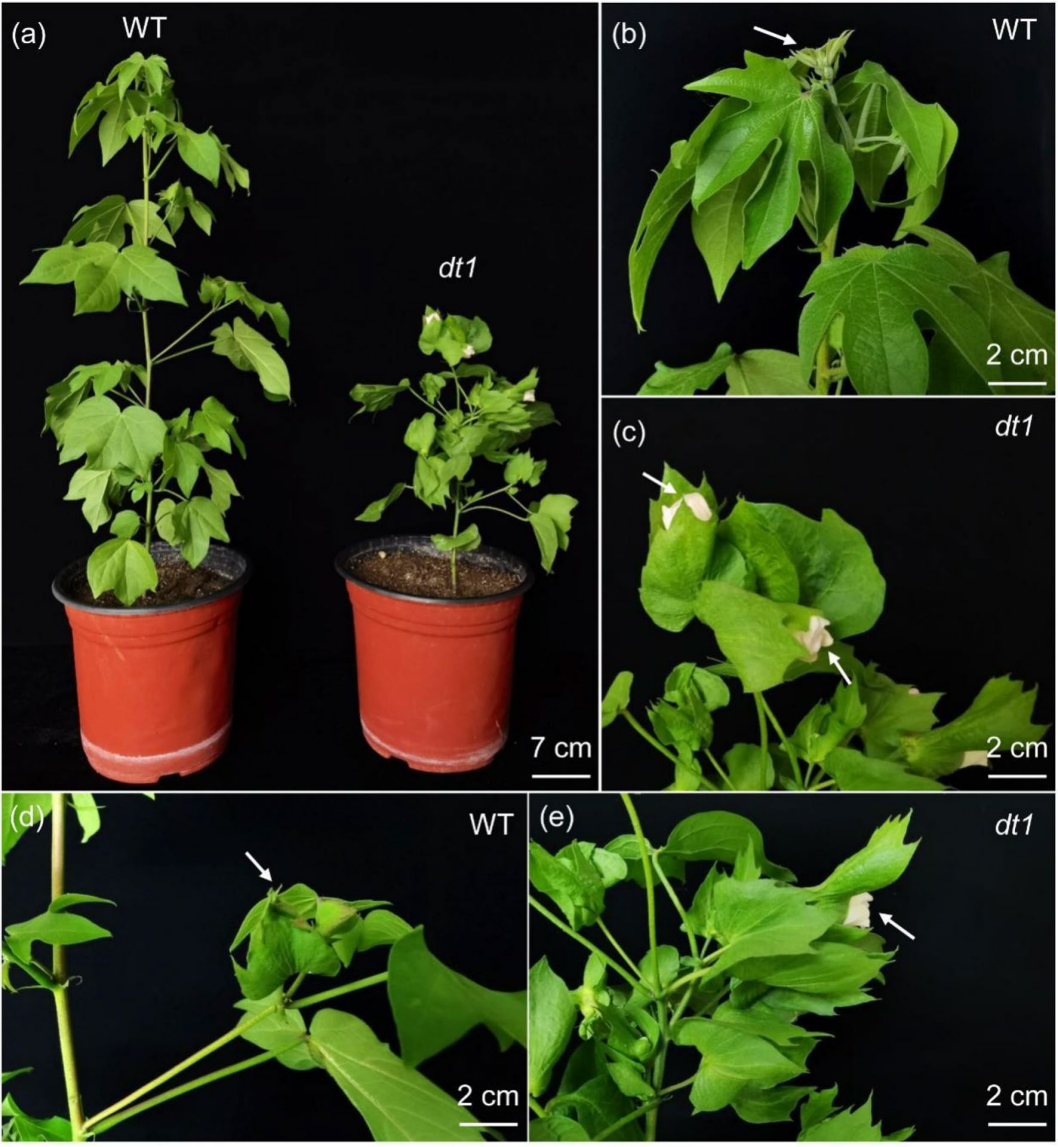
The phenotype of determinate growth and indeterminate growth in *dt1* and wild type Shixiya 1. **a** The plant architecture of the *dt1* mutant and the wild type Shixiya 1 (Scale bar = 7 cm) at the flower bud stage. **b**–**c** The destiny of the shoot apical meristem (SAM) in the *dt1* mutant and the wild type Shixiya 1. The white arrow indicates that the SAM became terminal flowers in the *dt1* mutant (**c**) compared with the young leaf formed in the wild type Shixiya 1 (**b**) (Scale bar = 2 cm). **d**–**e** The fruit branch of the *dt1* mutant and the wild type Shixiya 1. The white arrow indicates that the axillary buds of the lateral branches became flower buds in the *dt1* mutant (**e**) compared with the young leaf formed in the wild type Shixiya 1 (**d**) (Scale bar = 2 cm)

**Fig. 2:**
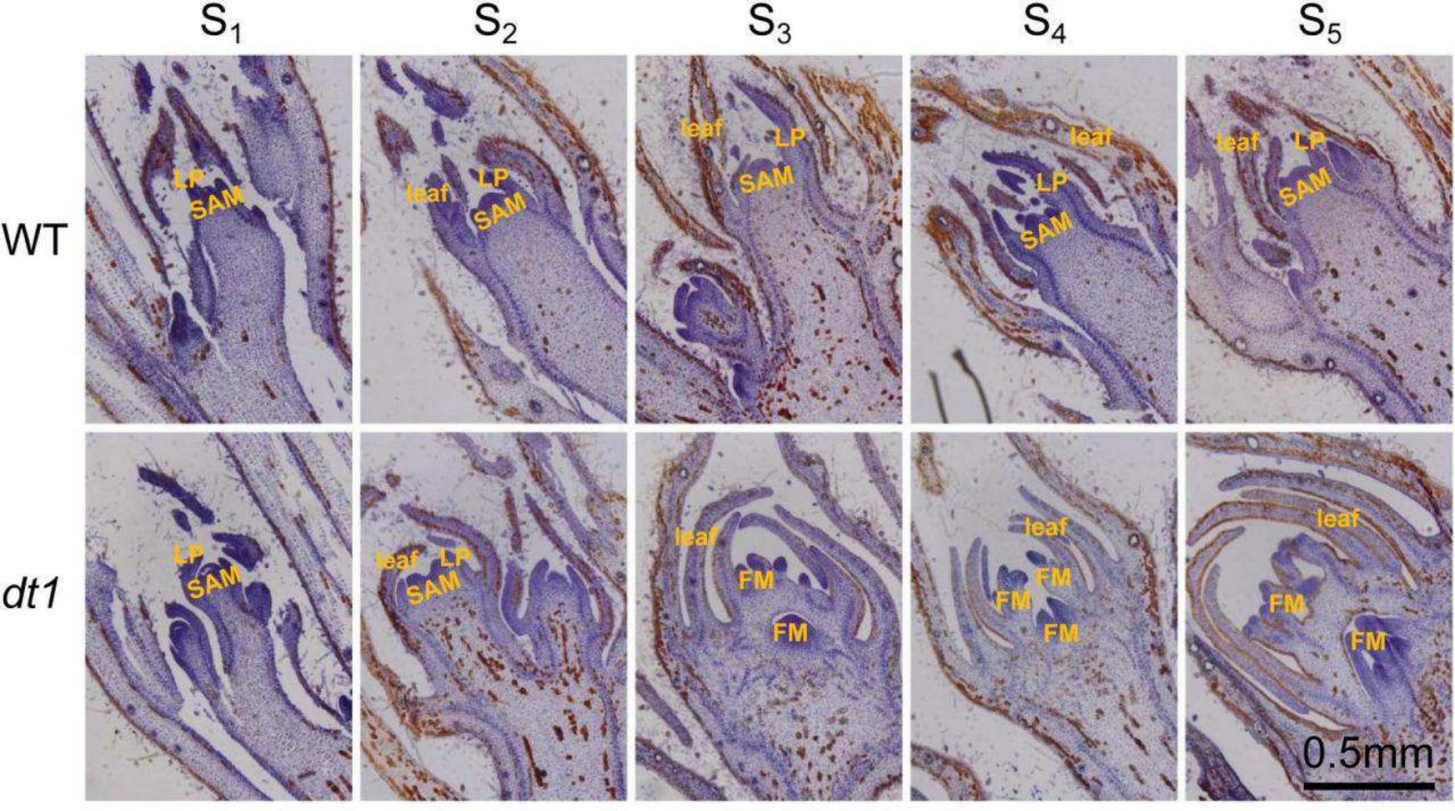
The long section analysis of the terminal buds. Microscopic observation of the terminal buds slices from one to five true leaf stages (from S1 to S5) in the *dt1* mutant and the wild type Shixiya 1. LP stands for leaf primordium, SAM stands for shoot apical meristem and FM stands for floral meristem. Scale bar = 0.5 mm

To study the plant architecture regulation in *G. arboreum*, the EMS mutation was made with the Shixiya 1 and a homozygous *determinate-growth 1* (*dt1*) plant was identified from the mutant population in M_1_–M_7_ plants. Compared with the wild type Shixiya 1, the SAM of the *dt1* mutant differentiated into a flower bud and then formed a terminal flower, and terminated indeterminate growth, causing an approximate half decrease in plant height (Fig. 1a–c). Meanwhile, axillary buds also developed directly into flowers, showing determinate growth phenotype (Fig. 1d, e). Therefore, we sliced the terminal buds of *dt1* mutant and WT to analyze the cytological difference during different development stages. The results showed that there was no significant difference in the terminal bud differentiation between the wild type Shixiya 1 and the *dt1* mutant at the stage of one-true leaf (S1) and two-true leaves (S2). However, at the three-true-leaf stage (S3), the terminal buds of the wild type Shixiya 1 continued to differentiate to new leaves and vegetative organs, remaining indeterminate growth, the differentiation ability of the terminal buds of the *dt1* mutant was significantly changed and formed the flower meristems and sepals gradually. In the following stages (S4 and S5), the clear leaf and stem primordium formed in the WT, while only a mature flower primordium formed in *dt1* (Fig. 2), which explained the reason for the formation of the terminal flower at the main stem in *dt1*.

## The* DT1* locus is located on a 0.85 Mb genomic region on chromosome A07 by BSA analysis and fine mapping

**Fig. 3:**
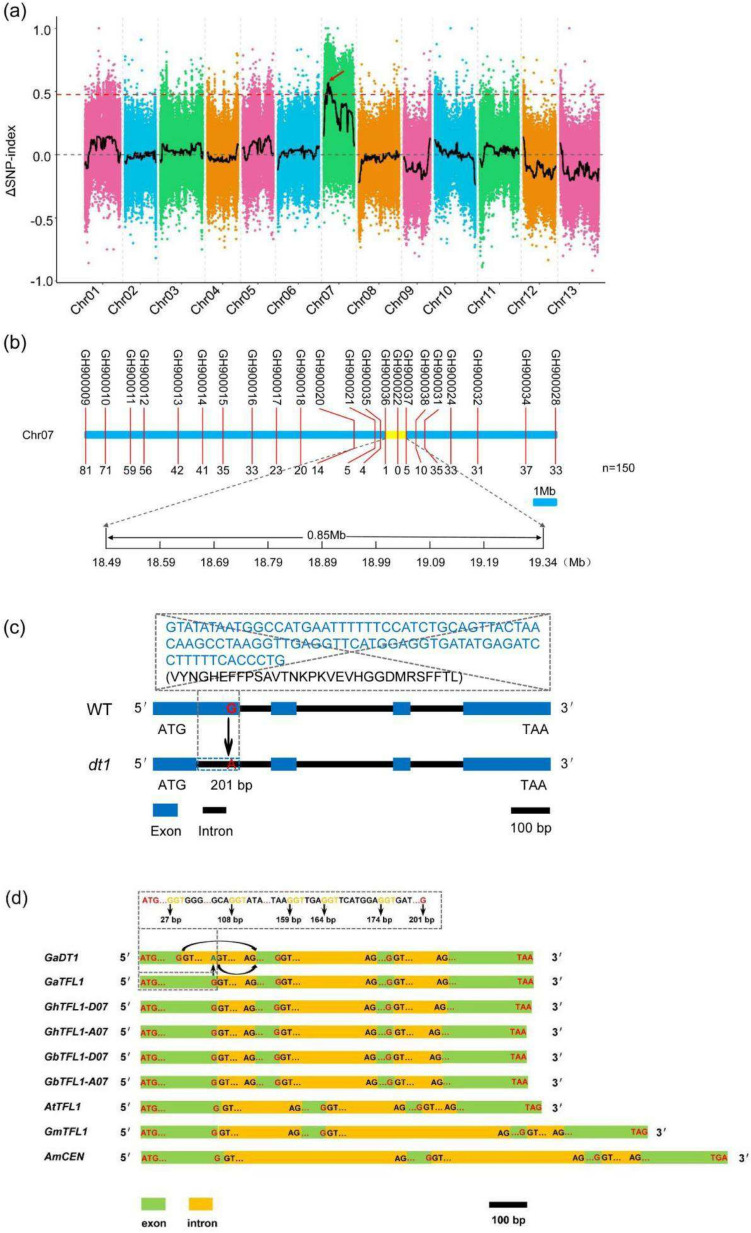
Fine mapping and cloning of *determinate growth 1*. **a** QTL analysis of the determinate growth phenotype in an F_2_ segregating population, the determinate growth locus was mapped on chromosome A07 from 7.31 Mb to 24.57 Mb. The red arrow indicates the only window with a ΔSNP value exceeding the 99% significance threshold confidence interval across the whole genome. The ΔSNP index (SNP index of the determinate growth bulk population subtracted from that of the indeterminate growth bulk population) and its 99% confidence interval are shown as black curves and red dotted lines (ΔSNP index > 0.5), respectively. **b** Genetic mapping of the determinate growth locus in a population with 150 F_2_ recessive individual plants by KASP. The number of recombinants was shown below the blue line. **c** The alternative 5′ splice site from GG to AG mutation in the first exon of *Ga07G1189* resulted in a 31 amino acids (VYNGHEFFPSAVTNKPKVEVHGGDMRSFFTL) deletion in the putative protein sequence. **d** The G/GT splice site in *TFL1* loci of other plants. In the gray dot box, the original G/GT splice site of *GaTFL1* is marked in red and other G/GT sites are marked in orange

To identify the causal locus for determinate growth of *dt1*, a *G. arboreum* line MZM 971 (indeterminate growth) and the *dt1* (determinate growth) were used as parents to construct a F_2_ population through hybridization and selfing. All F_1_ plants exhibited indeterminate growth, indicating that the determinate growth allele is recessive. The F_2_ population produced a 3:1 segregation ratio of indeterminate growth to determinate growth phenotypes (Table S2), indicating that a single locus controlled the determinate growth of *dt1*. Then, the bulked segregation analysis (BSA) was applied to identify the causal locus and the results showed that the ΔSNP index greater than 99% confidence intervals located on chromosome A07 from 7.31 Mb to 24.57 Mb (Fig. 3a) by aligning the sequence reads to the reference Shixiya 1 genome, demonstrating that the locus controlling the determinate growth is located on chromosome A07. Based on the above candidate interval, we first developed 22 KASP markers covering the genomic region from 5,744,881 bp (SNP marker GH900009) to 25,545,712 bp (SNP marker GH900028) (Table S1). Using these markers, we performed genotype testing in 150 recessive lines in the F_2_ generation population, and found that the determinate growth trait was closely linked to the 18.49–19.34 Mb interval, located at the 0.85 Mb genomic region between KASP markers SNP-GH900036 (pos.18496770) and SNP-GH900037 (pos.19341956) (Fig. 3b).

## Cloning of the *GaDT1*

There are 28 genes in the region from 18,496,770 to 19,341,956 on chromosome A07 of *G. arboreum* (Table S3) (*Cotton Functional Genomics Database: *https://cottonfgd.org/). We aligned the nucleic acids after sequencing of the 28 candidate genes between the two parents and found that *Ga07G1189* had an SNP in the first exon, *Ga07G1199* and *Ga07G1205* possessed SNPs in introns and 9 genes (*Ga07G1192*, *Ga07G1195*, *Ga07G1197*, *Ga07G1200*, *Ga07G1202*, *Ga07G1204*, *Ga07G1206*, *Ga07G1207*, *Ga07G1209*) had SNPs at the 2000 bp region upstream of the start codon, and there was no difference in the nucleotide sequences of other genes between the two parents (Fig. S1). Furthermore, semi-quantitative expression analysis indicated that only *Ga07G1189*, *Ga07G1195* and *Ga07G1207* displayed significant changes in transcription between Shixiya 1 and *dt1*, while other 9 genes didn’t show clear alteration in transcription during the floral differentiation (Fig. S2). In addition, we further analyzed the three candidate genes. *Ga07G1189* was annotated as “*terminal flower 1a* [*Gossypium hirsutum*]”, which was related to the determinate growth of many plants including *G. hirsutum* and *G. barbadense* (Chen et al. 2018; Kotoda et al. 2006; Njogu et al. 2020; Si et al. 2018). Moreover, the putative ORF from genomic DNA and RNA indicated that the single SNP represents an RNA 5′ splice site mutation from G to A (at the 201st position of the first exon) in the *dt1* mutant compared with the wild type Shixiya 1, and results in a 93-bp deletion from 108 to 200 bp in the coding sequence (CDS), as well as a 31 amino (VYNGHEFFPSAVTNKPKVEVHGGDMRSFFTL) deletion of *Ga07G1189* (*GaTFL1*) first exon in the *dt1* (Fig. 3c and Fig. S3). Regarding other two genes (*Ga07G1195* and *Ga07G1207*), no change in the mRNA sequence or amino acids sequence were identified except the transcription level. Therefore, we identified *Ga07G1189* as the potential candidate gene of *dt1.* Further, semi-quantitative expression analysis revealed that the *GaTFL1* transcription was significantly higher in the wild-type Shixiya 1 than that in the *dt1* mutant (Fig. S4).

The GU–AG rule is conserved in RNA splicing (Wang and Burge 2008). Through analysis of the transcripts and the alternative splicing in *Ga07G1189* between the WT and the *dt1* mutant, a novel splice site G/GT (boundary sequence of the first exon and intron) before the original splice site was identified to produce a shorter mature RNA and fewer amino acids, which was also confirmed by the sequencing of RT-PCR and genome PCR of *Ga07G1189* (Fig. S3), the primers used to amplify coding regions and full genomic regions of *Ga07G1189* gene were listed in Table S1. We also analyzed splicing profiles in *TFL1* of other plants, which confirmed the same and conserved 5′ splice site GU (or GT) in different *TFL1s*, and revealed that site G/GT was a novel splicing site of G/GT in *TFL1* loci (Fig. 3d). This finding suggested that other GT sites in genomic DNA which have not been recognized as splice sites previously might become the potential RNA splicing sites when plants encounter some specific stimuli to help plant change the function and survive.

## Silencing of *GaTFL1* results in terminal flowers similar to those in *dt1* phenotype plants

**Fig. 4:**
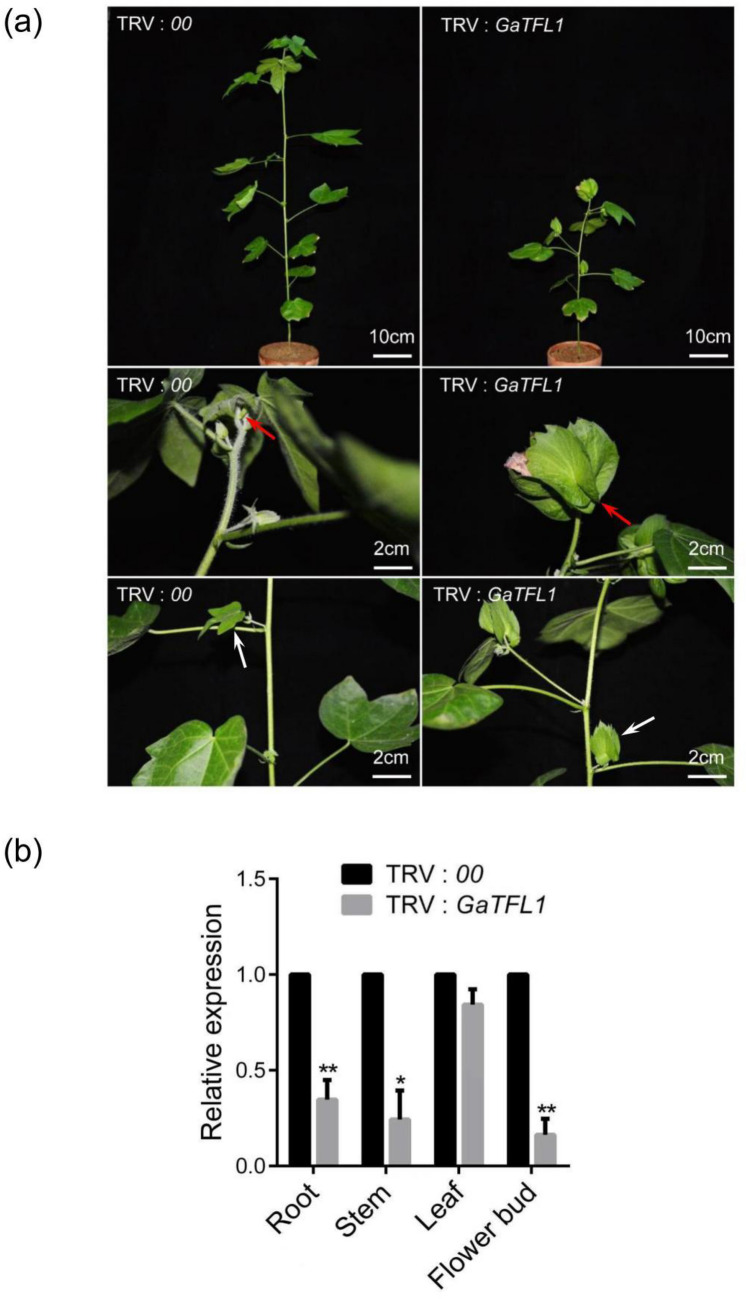
Silencing of *GaTFL1* results in terminal flower plants similar to *dt1* phenotype. **a** The phenotype of *Gossypium arboreum* line DQJ after silencing endogenous *GaTFL1* and blank vector infiltration plants. The red arrow indicates that the SAM became terminal flower in TRV: *GaTFL1* plant compared with the young leaf formed in blank vector control TRV: *00* plant, and the white arrow indicates that the axillary buds of the lateral branches became flower buds in TRV: *GaTFL1* plant compared with the young leaf formed in blank vector control TRV: *00* plant. **b** The level of *GaTEL1* transcript in different tissues of *GaTFL1*-silenced (TRV: *GaTFL1*) plants and the negative control (TRV: *00*). Two-tailed Student *t*-test was used for paired comparison of the *GaTFL1* gene in TRV: *GaTFL1* and TRV: *00* different tissues, respectively (**P < 0.01 or *P < 0.05)

To further test the function of *GaTFL1*, a 302 bp fragments including the first exon of *GaTFL1* was cloned and inserted into pTRV2 for VIGS. After 2 months postinfiltration, the TRV: *GaTFL1* consistently displayed terminal flowers, resulting in a determinate architecture compared to that in leaves infiltrated with a blank TRV: 00 vector (Fig. 4a), which was similar to the *dt1* phenotype plants, qRT-PCR analysis revealed that the expression of each respective *GaTFL1* in root, stem, leaf and flower bud tissues was much lower in *GaTFL1*-silenced plants compared with that in blank vector control TRV: *00* plants (Fig. 4b), indicating that *GaTFL1* may be the key gene for the indeterminate growth phenotype associated with the *dt1* locus.

## Analysis of differentially expressed genes between Shixiya 1 and *dt1*

**Fig. 5:**
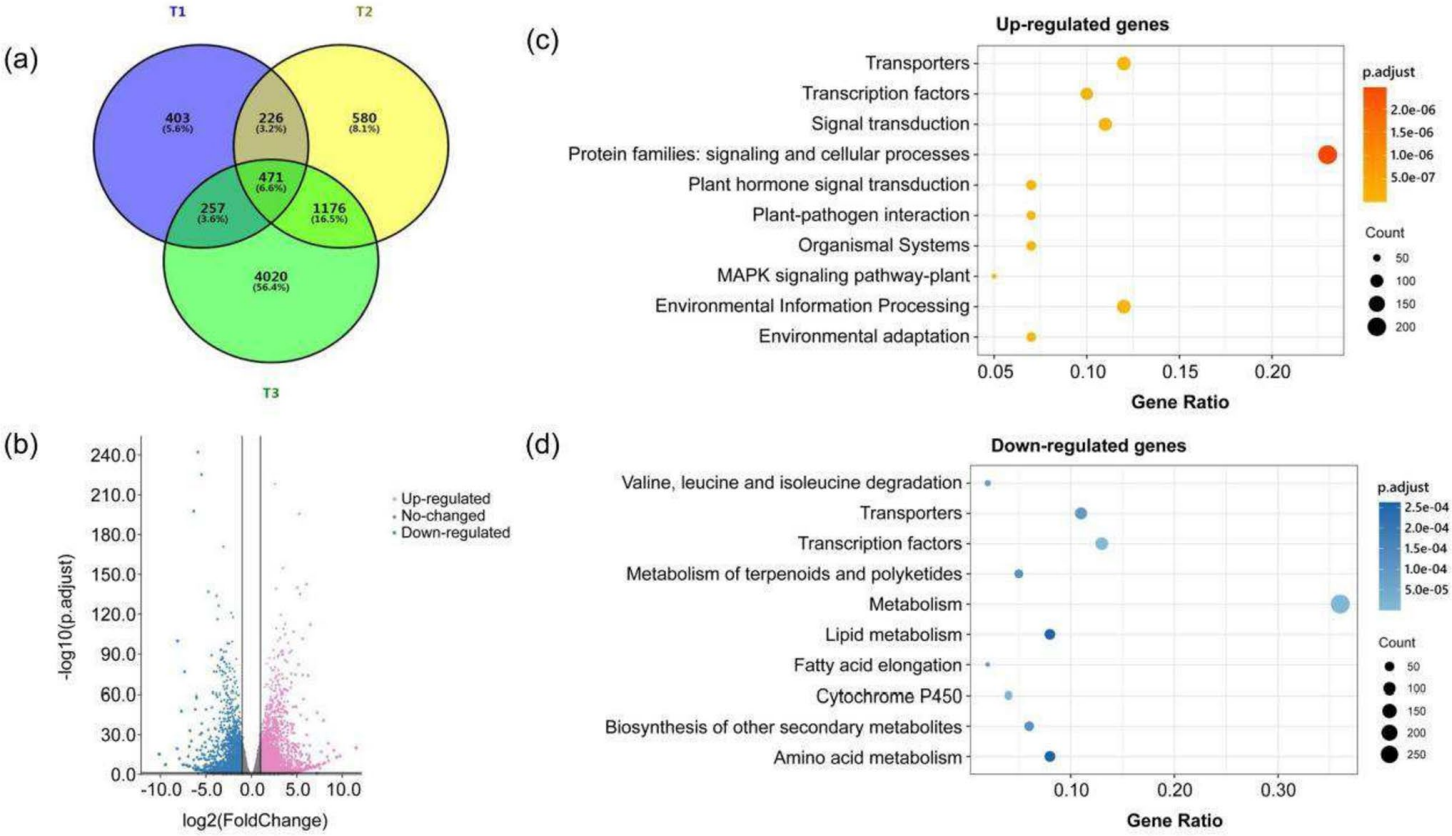
Transcriptomic comparison of the *dt1* mutant versus Shixiya 1. **a** Venn diagram showing the overlaps between the different stages of the *dt1* mutant and Shixiya 1. The number above each stage designation is the total transcripts detected in that stage(s). **b** Volcano plot of differentially expressed genes (DEGs) between the terminal bud of the *dt1* mutant and Shixiya 1 at T_3_ stage (flower budding stage, with visible flower buds). **c** The top 10 KEGG enriched pathways of up-regulated genes. **d** The top 10 KEGG enriched pathways of down-regulated genes. The top 10 KEGG enriched pathways are selected by p. adjust value sorting. Count, the bubble size, represents the number of enriched genes. GeneRatio represents the ratio of the number of DEGs corresponding to the pathway to the number of DEGs corresponding to the pathway database

To gain insight into the network and mechanism underlying the determinate growth development in *dt1*, RNA-Seq was performed with the terminal buds of Shixiya 1 and *dt1* at different stages T_1_ (seedling stage, three true leaves), T_2_ (the early stage of flower bud formation, no visible flower buds), and T_3_ (flower budding stage, with visible flower buds) to analyze the DEGs in the different stages with an adjusted *P*-value < 0.05 and an absolute value of fold change ≥ 2.0. In total, 7133 gene transcripts were identified, of which 471 were expressed at three stages, 5003 were stage-specific and 1659 were expressed at two stages. Strikingly, more stage-specific DEGs (4020) were identified in the T_3_ stage than that in the T_1_ (403) and T_2_ (580) stages (Fig. 5a), which was consistent with the phenotype at stage T3 when the *dt1* mutant formed the top flower resulting in the determinate growth phenotype. This indicates that many specific genes are involved in the regulation of the determinate growth in the T3 stage of *dt1*. A total of 5924 DEGs were identified in the T_3_ stage, including 3239 up-regulated and 2685 down-regulated genes (Fig. 5b and Table S4).

To reveal the causal genes and the underpinning resulting in the determinate growth of *dt1*, we performed KEGG analysis for the DEGs at the T_3_ stage. The KEGG enrichment analysis showed that both up-regulated and down-regulated genes related to transporters (e.g., MATE efflux family, Sugar transporter, ABC transporter) and transcription factors were enriched significantly (Fig. 5c, d). Seven MATE efflux family genes were up-regulated (Table S5), of which MATE1 and MATE2 genes are associated with the pleiotropic phenotypes of plants, such as leaf length, flowering time (Tiwari et al. 2014). Besides, 14 MATE efflux family proteins were down-regulated (Table S5), including one DTX1 protein and three ALF5 members, which positively regulated lateral root formation and increase root sensitivity to various compounds (Diener et al. 2001), and three TRANSPARENT TESTA 12-like proteins were involved in flavonoid transport (Marinova et al. 2007). Nine up-regulated and five down-regulated ABC transporters encoding genes were identified (Table S5), of which *ABCB19*, *ABCB1*, *ABCB21*, and *ABCB15* show redundant function and positively regulate auxin transport and plant height (Dudler and Hertig 1992). In addition, ten down-regulated genes involved in the sugar transport family, including five SWEET proteins and two SUC2-like proteins (Table S5), of which SWEET proteins may be involved in *Arabidopsis* inflorescence development (Chen et al. 2010, 2012; Yuan and Wang 2013). These results, to some extent, revealed the reasons for the different differentiation ability of terminal buds and the formation of terminal flowers in the *dt1* mutant and provided clues for the mechanisms of TFL1 in indeterminate growth regulation.

## Protein structure analysis reveals the functional divergence of TFL1 mutations in different *Nulliplex-branch* cottons

**Fig. 6:**
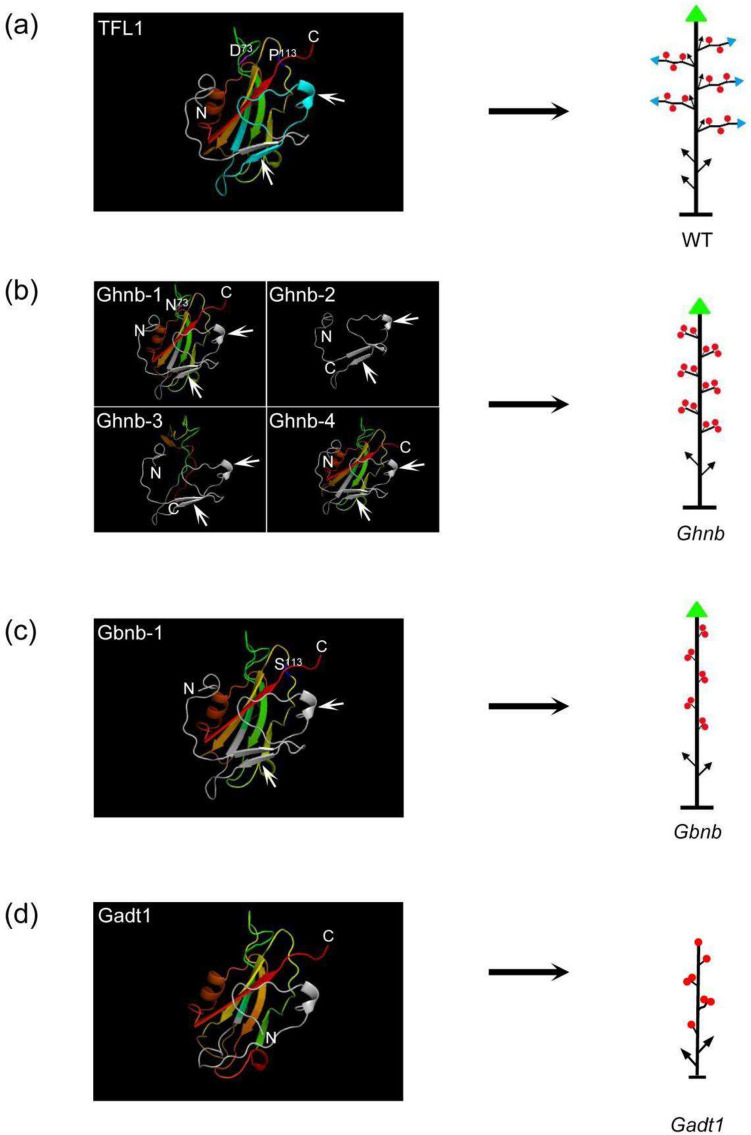
The protein structure and plant architecture analysis of the different types of *TFL1* alleles. **a** The 3D protein structure of TFL1 and plant architecture of indeterminate growth cotton. The amino acids of the first exon is marked in gray, of which the 31 amino acids deletion of *dt1* in the first exon is marked in light blue. **b** The 3D protein structure of Ghnb-1 to Ghnb-4 and plant architecture of *Ghnb* phenotype. The amino acids of the first exon is marked in gray, and the mutation of D73N amino acid is marked in purple. **c** The 3D protein structure of Gbnb-1 and plant architecture of *Gbnb* phenotype. The amino acids of the first exon is marked in gray, and the mutation of P113S amino acid is marked in bule. **d** The 3D protein structure of Gadt1 and plant architecture of *Gadt1* phenotype. Amino (N) and carboxy (C) termini are labeled. White arrows in (**a**), (**b**) and (**c**) represent the missing β-sheets in (**d**). Green triangles represent monopodial SAMs, blue triangles represent sympodial SAMs, black arrows represent vegetative branches, and red circles represent bolls

The previous research has identified several TFL1 mutants in different *Nulliplex-branch* (*nb*) mutants such as *Ghnb-1*, *Ghnb-2*, *Ghnb-3*, *Ghnb-4*, *Gbnb-1* (Chen et al. 2018; Si et al. 2018), which all display indeterminate growth of main stem and short fruit branches that terminate in a cluster of two or more flowers (Fig. 6a–c). The *Gadt1* displays determinate growth of main stem with a terminal flower and causes one or more clustered flowers with elongated pedicels to be attached directly to the main stem (Fig. 6d), whose determinate growth habit of main stem differed significantly from the other *nb* lines (Fig. 6a–c). The difference in plant architecture phenotypes between *Gadt1* and other *nb* mutants indicates the potential protein functional divergence of different TFL1 mutations. For *Ghnb* lines, four mutations within the CDS region were detected, namely, a single nucleotide mutation from G to A at position 217 in exon 2, causing a non-synonymous amino acid change, D73N (designated as Ghnb-1), a single T deletion at position 194 in exon 1, a two nucleotide deletion at positions 254–255 in exon 2, and a single T deletion at position 491 in exon 4 (designated as Ghnb-2 to Ghnb-4, respectively). For *Gbnb* line, a single nucleotide mutation from C to T at position 337 in exon 4, caused a non-synonymous amino acid change, P113S (designated as Gbnb-1) (Chen et al. 2018). For Gadt1, a single nucleotide mutation from G to A at position 201 in exon 1, changed the 5′ splice site and deleted 31 amino acid (Fig. 3c). To explore the protein tertiary structure differences among them, we used Phyre2 to predict the 3D structure of the mutational TFL1 in the different *nb* lines, and found that there was no significant difference in the corresponding protein structures of the first exon of Ghnb-1, Ghnb-2, Ghnb-3, Ghnb-4 and Gbnb-1 compared with the WT, harboring the unabridged β-sheets and pocket (Fig. 6a–c). However, the novel mutant Gadt1 displayed a novel variation of 3D structure for the truncated protein, showing two β-sheets missing (Fig. 6a,d), which may damage the intact pocket and the key function of TFL1 protein to result in the change in both main stem and lateral branches.

## Discussion

The domestication of crops prefers more determinate architectures with compact growth habits and synchronous flowering. Therefore, comprehending the underlying mechanism leading to the difference between indeterminate and determinate growth can benefit crop improvement. Generally, indeterminate plants produce inflorescence on flanks, while determinate plants generate a flower or inflorescence at the top. Many factors have been identified involved in the inflorescence and plant architecture regulation, among which the FT-TFL1 model has been identified as the key factor with antagonistic functions in inflorescence and plant architecture controlling (Teo et al. 2014; Wickland and Hanzawa 2015).

## A single nucleotide mutation alters the splicing pattern in the *TFL1* locus

Cotton species displays naturally indeterminate growth. Under favorable conditions, both of the main stem and the lateral branches remain indeterminate growth (Fig. 6a). However, several determinate growth lines such as Caozao-3 (*nb*-type upland cotton), Xinhai-18, (*nb*-type Pima cotton) and a modern ELS cultivars (extra-long-staple cotton (AD)2, *G. barbadense* L.) have been recognized in tetraploid lines, and the determinate growth trait can be stably inherited. Further studies also explored the detailed nucleotide in the above *nb* mutants (Chen et al. 2018; Si et al. 2018). In the present study, we also identified a similar *determinate growth 1* (*dt1*) mutant in diploid line Shixiya 1 (*G. arboreum*) with the EMS mutagenesis approach. The gene cloning revealed that the determinate growth phenotype in all the above lines is controlled by the various mutations in the same *TFL1* locus, supporting the vital role of TFL1 in plant architecture regulation. So, we analyzed the mutation sites in the *TFL1* locus of *G. arboreum* and uncovered that a novel single nucleotide mutation from G to A at the 201st position changed the splicing site of the first exon/intron and produced a shorter exon, which generated a truncated protein with loss of function. Moreover, *Gadt1* also showed some different plant architecture changes compared with other *nb* cottons. The prediction and analysis of 3D structure of the mutational TFL1 in the different *nb* lines revealed a novel variation of 3D structure for the truncated protein of Gadt1 (Fig. 6d), suggesting that different domains or amino acids of TFL1 may have distinct function to contribute to the plant architecture regulation. Further, we also analyzed the splicing sites in other *TFL1* genome sequences from *Arabidopsis thaliana*, *Glycine max*, and *Antirrhinum majus*. These findings indicated that G/GT but not A/GT is a recognizable site of RNA splicing in the plant *TFL1* loci, which provided potential targets for the *TFL1* gene editing by CRISPR-Cas9 technology as well as some clues for the gene alternative splicing sites study in the plant. However, several other G/GT sites were also found around the original splice G/GT (201st bp) of *TFL1* (Fig. 3d), why they are not recognized in *dt1* by the spliceosome is still unclear. Understanding the detailed mechanisms of interaction between spliceosome and *TFL1* pre-mRNA with the Hi-C technology, specific site mutation technology et al. would contribute to explore the underpinning for alternative splicing in *GaDT1*. These results indicated the diversity and specificity of alternative splicing mechanisms and spliceosomes in cells.

## The mechanisms of TFL1 in the indeterminate growth of the plant

Based on previous research, it was proposed that the balance of determinate and indeterminate growth in all plants is controlled by the balance of the activities of the FT and TFL1 homolog gene products (Lifschitz et al. 2014, 2006; Pnueli et al. 1998; Shalit et al. 2009). The mutation of TFL1 alters the inflorescence architecture and results in the determinate growth in different plants, and contributes to the crop yield, mechanical harvest, and human domestication such as cotton, tomato, and soybean (Chen et al. 2018; Pnueli et al. 1998; Tian et al. 2010). While the underlying mechanism that TFL1 regulates inflorescence and plant architecture is still ambiguous. In general, TFL1 and FT compete for the interaction with FD to involve the floral organ and inflorescence formation as mobile factors (Benlloch et al. 2007; Hanano and Goto 2011; Liljegren et al. 1999; Ratcliffe et al. 1999). Interestingly, both of them also bind TFL1 and FT are distinct through only a little part of nonconservative amino acid changes, supporting their common binding ability with FD and lipid di 18:1-PC (Chen et al. 2018), although they display antagonistic roles (Ho and Weigel 2014; Ahn et al. 2006). A single amino acid substitution can convert FT into TFL1 and vice versa (Ho and Weigel 2014; Ahn et al. 2006; Hanzawa et al. 2005; Pin et al. 2010). So, minor amino acids change might influence the TFL1 function obviously, which may be realized through the delicate adjustment on the function domain or the 3D structure of protein. The results from this study further provide valuable clues to elucidate the complex and fine interaction between FT and TFL1 in the inflorescence regulation. In both FT and TFL1, a large central β-sheet and a smaller β-sheet comprise the important pocket motif, which are resulted from the first exon (Ahn et al. 2006). Further structural analysis of TFL1 suggests that its pocket is able to accommodate phosphoryl groups but not phospholipid, suggesting the potential signaling mediation by TFL1 via their association with phosphorylated proteins (Pnueli et al. 2001). The previous interaction and activity inhibition between a mouse PEBP and the kinase Raf1 also supported the possibility (Yeung et al. 1999). Therefore, the GaDT1 seems disturb the upstream signaling transduction in the formation of SAM and axillary meristem simultaneously to affect the plant architecture. Through some technologies such as Nuclear Magnetic Resonance (NMR) and Immunoprecipitation-Mass Spectrometry (IP-MS), the specific interactors with the pocket or β-sheets in the first exon could be identified, which would be very beneficial to reveal the TFL1 function mechanism and explain the difference between different TFL1 mutants.

**Fig. 7:**
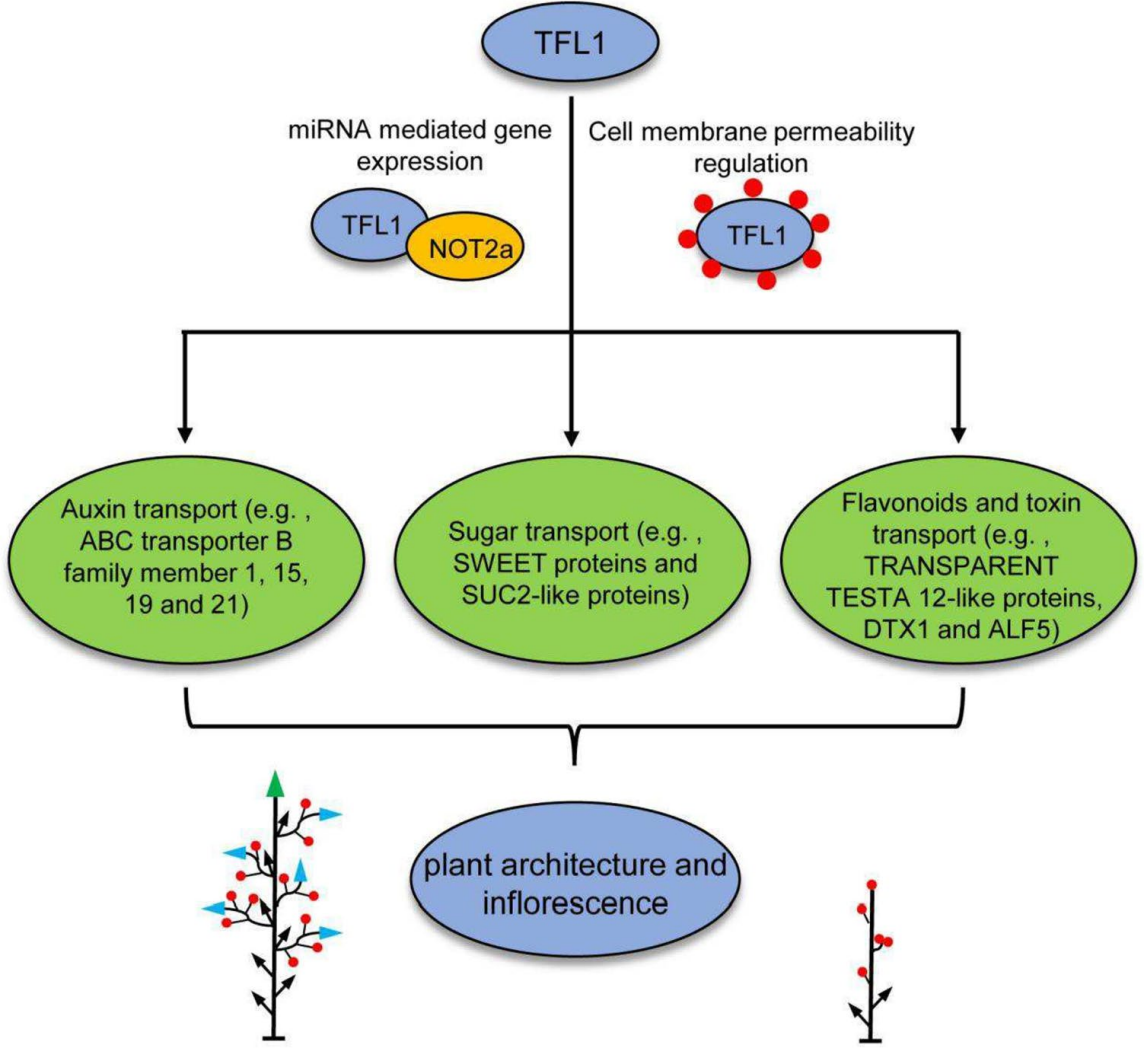
The hypothesis model of functional mechanism of TFL1 in plant architecture regulation. TFL1 associated complex are involved in the transport and translocation of sugar, key metabolites (e.g., flavonoids, toxin) or phytohormones (e.g., auxin) for the SAM tissue development. In these processes, TFL1 interacts with NOT2a or lipid di 18:1-PC to regulate the expression and function of genes encoding transporters responsible for auxin, sugar, flavonoids and toxin through epigenetic modification pathway or direct structure modification. With this mechanism, TFL1 function upstream of the auxin, sugar, flavonoids and toxin transport pathways to coordinate the vegetative and reproductive development and plant architecture. (Red circles around TFL1 represent lipid di 18:1-PC; Green triangles represent monopodial SAMs, blue triangles represent sympodial SAMs, black arrows represent vegetative branches, and red circles represent bolls)

In a word, the exhaustive study for every amino acid or nucleotide, and the unidentified regions of protein would be necessary to explore the function of TFL1/FT, as well as the underlying mechanism for the competition between FT and TFL1 for FD. In cucumber, CsTFL1 competes with CsFT for interaction with a homolog of the miRNA biogenesis gene, CsNOT2a. Then, the complex interacts with CsFDP (Wen et al. 2019). These indicated that TFL1 may be involved in different molecular and physiological pathways. Even so, the detailed downstream pathway of TFL1 is unclear. Here, we performed an RNA-Seq with the terminal SAM tissues in *dt1* mutant and WT to propose a model (Fig. 7). After the DEGs analysis, some transporters responsible for the auxin, sugar, and flavonoids are enriched downstream of TFL1 in the determinate growth regulation (Table S5). Massive literature reported that auxin and sugar play important roles in SAM to regulate inflorescences and plant architecture establishment (Vernoux et al. 2010; Xing et al. 2015; Yoon et al. 2021; Zhu and Wagner 2020), indicating that TFL1 associated complex or functional models are involved in the transport and translocation of key metabolites or phytohormones for the SAM tissue development. The previous direct interaction between TFL1 and NOT2a or lipid (Chen et al. 2018; Wen et al. 2019) suggested that TFL1 might regulate the transporters’ expression and function through epigenetic modification (i.e., miRNA mediated gene regulation) or cell membrane permeability mediation, bridging the link between TFL1 and the transport of key materials such as auxin, sucrose and flavonoids to regulate plant architecture and inflorescence (Fig. 7). In previous studies, the FT-TFL1 model, auxin, and sugar are known as the important factors in inflorescence and plant architecture, but the correlation among them is always unclear. The results of this study demonstrated that TFL1 may function upstream of the auxin and sugar pathways by mediating their transport to coordinate the vegetative and reproductive development from the SAM (Fig. 7). Some genetic research or biochemistry experiments with immunoprecipitation of TFL1 antibody would be helpful to elucidate the detailed relationship between TFL1 and transporters.

## Conclusion

The *dt1* mutant in *G. arboreum* was identified through EMS mutagenesis, which displayed determinate growth in both of the SAM and axillary buds with a terminal flower. The map-based cloning of the *DT1* locus showed a single nucleotide mutation from G to A at the 201st position changed the 5′ RNA splice site and deleted 31 amino acid in the first exon of *GaTFL1*, which is a novel mutation site different from the Ghnb-1, Ghnb-2, Ghnb-3, Ghnb-4 and Gbnb-1. Comparative transcriptomic RNA-Seq analysis indicated that GaTFL1 may function upstream of auxin, sugar, and flavonoids. Taken together, our results contribute to explore the diversity and specificity of alternative splicing mechanisms and spliceosomes in cells and provide novel clues for the TFL1 mechanism in plant development regulation and research strategies for plant architecture improvement.

### Supplementary Information

Below is the link to the electronic supplementary material.

Supplementary file1 Table S1. The primer in this study. Table S2. Chi-square test and genetic analysis of F2 population (DOCX 20 KB)

Supplementary file2 Table S3. The list of the candidate genes (XLSX 11 KB)

Supplementary file3 Table S4. The differentially expressed genes of the *dt1* mutant versus Shixiya 1 at the different stages (XLSX 8507 KB)

Supplementary file4 Table S5. The classification of transporters in KEGG enrichment pathway (XLSX 21 KB)

Supplementary file5 Figure S1. Integrative Genomics Viewer (IGV) analysis for the resequencing data of the 28 candidate genes. The SNP sites between the parent and the reference genome were marked by red lines. Figure S2. Semi-quantitative RT-PCR analysis of the candidate genes between the *dt1* mutant and the wild-type Shixiya 1. The red dot box suggests the difference between the *dt1* and the WT. Figure S3. Sequence alignment of *Ga07G189* between the *dt1* mutant and wild type Shixiya 1. **a** Coding sequence (CDS) alignment of *Ga07G189* between the *dt1* mutant and Shixiya 1, a 93-bp deletion was observed in the *dt1* mutant, **b** Full genomic sequence alignment of *Ga07G1189* between the *dt1* mutant and Shixiya 1, a SNP (G to A) was observed in the dt1 mutant. The CDS sequence and the genome sequence of Ga07G1189 were obtained by RT-PCR and genome PCR, and used here, respectively. Figure S4. Semi-quantitative RT-PCR analysis of the *GaTFL1* between the *dt1* mutant and the wild-type Shixiya 1 (DOCX 5075 KB)

## Data Availability

All the data sets generated during the current study are available in the NCBI Sequence Read Archive (SRA) under project number PRJNA989558 for BSA-seq and PRJNA989394 for RNA-seq.
