# Supplementary material for: A novel single nucleotide mutation of TFL1 alters the plant architecture of Gossypium arboreum through changing the pre-mRNA splicing
Source: Plant Cell Rep. 2023 Dec 29;43(1):26. doi: 10.1007/s00299-023-03086-7 (PMC10754752; doi:10.1007/s00299-023-03086-7)
Supplement: Supplementary file 1 — Supplementary file1 Table S1. The primer in this study. Table S2. Chi-square test and genetic analysis of F2 population (DOCX 20 KB) [file 299_2023_3086_MOESM1_ESM.docx]

**Table S1.** The primer in this study

| Primer | Sequence（5΄→3΄） | Application |
| --- | --- | --- |
| *Ga07G1189* QF  *Ga07G1189* QR  *Ga07G1192* QF  *Ga07G1192* QR  *Ga07G1195* QF  *Ga07G1195* QR  *Ga07G1197* QF  *Ga07G1197* QR  *Ga07G1199* QF  *Ga07G1199* QR  *Ga07G1200* QF  *Ga07G1200* QR  *Ga07G1202* QF  *Ga07G1202* QR  *Ga07G1204* QF  *Ga07G1204* QR  *Ga07G1205* QF  *Ga07G1205* QR  *Ga07G1206* QF  *Ga07G1206* QR  *Ga07G1207* QF  *Ga07G1207* QR  *Ga07G1209* QF  *Ga07G1209* QR  His3 QF  His3 QR  *Ga07G1189* F  *Ga07G1189* R  GH900009F_FAM  GH900009F_HEX  GH900009R  GH900010F_FAM  GH900010F_HEX  GH900010R  GH900011F_FAM  GH900011F_HEX  GH900011R  GH900012F_FAM  GH900012F_HEX  GH900012R  GH900013F_FAM  GH900013F_HEX  GH900013R  GH900014F_FAM  GH900014F_HEX  GH900014R  GH900015F_FAM  GH900015F_HEX  GH900015R  GH900016F_FAM  GH900016F_HEX  GH900016R  GH900017F_FAM  GH900017F_HEX  GH900017R  GH900018F_FAM  GH900018F_HEX  GH900018R  GH900020F_FAM  GH900020F_HEX  GH900020R  GH900021F_FAM  GH900021F_HEX  GH900021R  GH900035F_FAM  GH900035F_HEX  GH900035R  GH900036F_FAM  GH900036F_HEX  GH900036R  GH900022F_FAM  GH900022F_HEX  GH900022R  GH900037F_FAM  GH900037F_HEX  GH900037R  GH900038F_FAM  GH900038F_HEX  GH900038R  GH900031F_FAM  GH900031F_HEX  GH900031R  GH900024F_FAM  GH900024F_HEX  GH900024R  GH900032F_FAM  GH900032F_HEX  GH900032R  GH900034F_FAM  GH900034F_HEX  GH900034R  GH900028F_FAM  GH900028F_HEX  GH900028R  pTRV2-*GaTFL1* F  pTRV2-*GaTFL1* R | CCTCTTCAAGCAGAAAGGCAGGC  GCGTCTTCTAGCAGCTGTTTCCC  TGGCGCCGCTTAGATAAACA  TTTGTATGCCCCTCCACACG  ACTTGCGATTGTGGGAGGTT  GGAGCAAGCGATACAGAGGG  GGCCAACCCAATTCCCAAGA  GGAGCAAAAGAAGTGGCATGG  GGTGAGAGTCAAGCCTGGAG  AACAGCAGCCTTCATAGGGG  AGATCCTTTGACCGCACTGG  TGGAAGGCCTCTGATCTCCA  CTGGACGGGATAGAGTTGGC  GTGCCGCTAATCTCCACCTT  TTCGACGAAGATTGACTACAGCA  TACACTTGCCGTTACTCTCTGC  ACCTCTGCCAAAGCCCTTAC  TTCAGGCGTCCTACTGGAGA  TCAAGAACACGTGGGAAACTTG  TCCTTCTTCGATGTTGTCCAGG  TTCCCTACTGAACCTAGCAGA  ACAGGAATGACATTTTCTCTACAAC  CGGGGCTTTTGTTTGAGAGC  TGAACAGTCCCAAGTGCCAT  TCAAGACTGATTTGCGTTTCCA  GCGCAAAGGTTGGTGTCTTC  ATGGCAAAACTGTCAGATCC  TTAGCGTCTTCTAGCAGCTG  GAAGGTGACCAAGTTCATGCTGATCATGTTCGTTGCCTTCGTAATG  GAAGGTCGGAGTCAACGGATTGATCATGTTCGTTGCCTTCGTAATC  AGGAAGGAGAGATGGACGCTGACAT  GAAGGTGACCAAGTTCATGCTTCTATATCCTTCCCTTTCCCTAAACTATC  GAAGGTCGGAGTCAACGGATTTCTATATCCTTCCCTTTCCCTAAACTATT  ACGGCTCCGCATTATCTTCATAATCA  GAAGGTGACCAAGTTCATGCTGCCTTTTAATTTTAGTGATTTGGGTCTG  GAAGGTCGGAGTCAACGGATTGGCCTTTTAATTTTAGTGATTTGGGTCTA  CAGTATGACTTGTGTAGGGCCCAAAT  GAAGGTGACCAAGTTCATGCTTCTAGGTACTGTCACTCTGTTGAACTCCC  GAAGGTCGGAGTCAACGGATTTCTAGGTACTGTCACTCTGTTGAACTCCT  GAGGGTTTTGTCTTATGACTTCACTG  GAAGGTGACCAAGTTCATGCTCAGCAGTTGCTAACAAGTCCGATTAT  GAAGGTCGGAGTCAACGGATTCAGCAGTTGCTAACAAGTCCGATTAC  TGACTAGTAGAAGGAGGAATCAGAAGATTG  GAAGGTGACCAAGTTCATGCTTGGGCTATATTGGTTAGAACTAGGAAGAAG  GAAGGTCGGAGTCAACGGATTTGGGCTATATTGGTTAGAACTAGGAAGAAA  TGTCTTGATGTGTTGATATAACTCCTTTTG  GAAGGTGACCAAGTTCATGCTGGTGAAGGTGGAGGTAGGCAAAAT  GAAGGTCGGAGTCAACGGATTGGTGAAGGTGGAGGTAGGCAAAAG  TCATACCCGTCACACATCGAACCTAA  GAAGGTGACCAAGTTCATGCTTGGGTCTAGTCTTCCGTCCAAAGTT  GAAGGTCGGAGTCAACGGATTTGGGTCTAGTCTTCCGTCCAAAGTC  GCCTAAACCCTCTCATATTTCGAGCA  GAAGGTGACCAAGTTCATGCTAATTAAATCAATGCCATGAATCTTCTTTTC  GAAGGTCGGAGTCAACGGATTAATTAAATCAATGCCATGAATCTTCTTTTT  CCCTGTATGGTTGGAAATGTAGAACT  GAAGGTGACCAAGTTCATGCTCGATAATGCTCTGTCACTCTGTTTCA  GAAGGTCGGAGTCAACGGATTCGATAATGCTCTGTCACTCTGTTTCG  TCCATGTAACACCCCTAACCCGTATC  GAAGGTGACCAAGTTCATGCTCCAATTTCGAATGGAACTGGATGATA  GAAGGTCGGAGTCAACGGATTCAATTTCGAATGGAACTGGATGATG  TCCATGCCTTCCTTCAAGTCTTTTTC  GAAGGTGACCAAGTTCATGCTATCTGTCGTGACTGAGAAGGAACCA  GAAGGTCGGAGTCAACGGATTTGTCGTGACTGAGAAGGAACCG  TGTGCAGCAACTATCCATTCAGGACT  GAAGGTGACCAAGTTCATGCTGCGTGGCATTTACTAGGATTTGAAGT  GAAGGTCGGAGTCAACGGATTCGTGGCATTTACTAGGATTTGAAGC  CGAGTTTAATTCTTTTCCCTTGCATCA  GAAGGTGACCAAGTTCATGCTTAGTTTGTTGGGCTACATACCAAGC  GAAGGTCGGAGTCAACGGATTTAGTTTGTTGGGCTACATACCAAGT  TCATTTCCAATTGACTAACATCATTG  GAAGGTGACCAAGTTCATGCTCTTCTAGGCTTGGCACTTGGTGTT  GAAGGTCGGAGTCAACGGATTCTTCTAGGCTTGGCACTTGGTGTC  TGCTCTAACTAGCCATTCCAACCAAT  GAAGGTGACCAAGTTCATGCTAGCCATTGATGTTCCTGGTTTGTT  GAAGGTCGGAGTCAACGGATTCAGCCATTGATGTTCCTGGTTTGTA  CTGACAGAAATGATCAGAAACAAAA  GAAGGTGACCAAGTTCATGCTGGTCAATGTGGTGGCTGACACTCTA  GAAGGTCGGAGTCAACGGATTGTCAATGTGGTGGCTGACACTCTG  TCGCTCTCAAATCAGTCATCGCTCT  GAAGGTGACCAAGTTCATGCTTAGCTCCTTAGAGGGAGTGGGTTTC  GAAGGTCGGAGTCAACGGATTGATAGCTCCTTAGAGGGAGTGGGTTTT  TCATTTCCTCTATTATATTCGCTTTGTCCA  GAAGGTGACCAAGTTCATGCTTTGCTCTTGTTTATGATCTTGTTGAACTT  GAAGGTCGGAGTCAACGGATTTTGCTCTTGTTTATGATCTTGTTGAACTC  CACTAATGGTGGTGATTTCTGGTTTGA  GAAGGTCGGAGTCAACGGATTTTGGACCTATTCCAAAACTGCTCAAT  GAAGGTGACCAAGTTCATGCTTGGACCTATTCCAAAACTGCTCAAC  TTCTACGTGTACGACCATTGGGAGTG  GAAGGTGACCAAGTTCATGCTGGCCAAATTGAAAGCGATTGTAAC  GAAGGTCGGAGTCAACGGATTGGCCAAATTGAAAGCGATTGTAAG  TCCAATCTCGTTCCTAACTTTTTCTGG  GAAGGTGACCAAGTTCATGCTGCAACTAAACGCACCACCCATTAC  GAAGGTCGGAGTCAACGGATTGCAACTAAACGCACCACCCATTAT  TAATATTTCCCTCCCTAGCCCCTCCT  ctgtgagtaaggttaccgaattcCCTTGGCATTTCGTAGTTCA  tcgagacgcgtgagctcggtaccGAGTGATTGGGGATGTTATTGA | RT-qPCR  RT-qPCR  RT-qPCR  RT-qPCR  RT-qPCR  RT-qPCR  RT-qPCR  RT-qPCR  RT-qPCR  RT-qPCR  RT-qPCR  RT-qPCR  RT-qPCR  Gene clone  KASP mark  KASP mark  KASP mark  KASP mark  KASP mark  KASP mark  KASP mark  KASP mark  KASP mark  KASP mark  KASP mark  KASP mark  KASP mark  KASP mark  KASP mark  KASP mark  KASP mark  KASP mark  KASP mark  KASP mark  KASP mark  KASP mark  VIGS |

**Table S2.** Chi-square test and genetic analysis of F_2_ population

| F2 population | Sum | The indeterminate growth | The determinate growth | Expected ratio | χ^2^ (1, 0.05) =3.84 |
| --- | --- | --- | --- | --- | --- |
| MZM971 × *dt1* | 336 | 257 | 79 | 3:1 | 0.40 |
