# Supplementary material for: A novel single nucleotide mutation of TFL1 alters the plant architecture of Gossypium arboreum through changing the pre-mRNA splicing
Source: Plant Cell Rep. 2023 Dec 29;43(1):26. doi: 10.1007/s00299-023-03086-7 (PMC10754752; doi:10.1007/s00299-023-03086-7)
Supplement: Supplementary file 5 — Supplementary file5 Figure S1. Integrative Genomics Viewer (IGV) analysis for the resequencing data of the 28 candidate genes. The SNP sites between the parent and the reference genome were marked by red lines. Figure S2. Semi-quantitative RT-PCR analysis of the candidate genes between the dt1 mutant and the wild-type Shixiya 1. The red dot box suggests the difference between the dt1 and the WT. Figure S3. Sequence alignment of Ga07G189 between the dt1 mutant and wild type Shixiya 1. a Coding sequence (CDS) alignment of Ga07G189 between the dt1 mutant and Shixiya 1, a 93-bp deletion was observed in the dt1 mutant, b Full genomic sequence alignment of Ga07G1189 between the dt1 mutant and Shixiya 1, a SNP (G to A) was observed in the dt1 mutant. The CDS sequence and the genome sequence of Ga07G1189 were obtained by RT-PCR and genome PCR, and used here, respectively. Figure S4. Semi-quantitative RT-PCR analysis of the GaTFL1 between the dt1 mutant and the wild-type Shixiya 1 (DOCX 5075 KB) [file 299_2023_3086_MOESM5_ESM.docx]

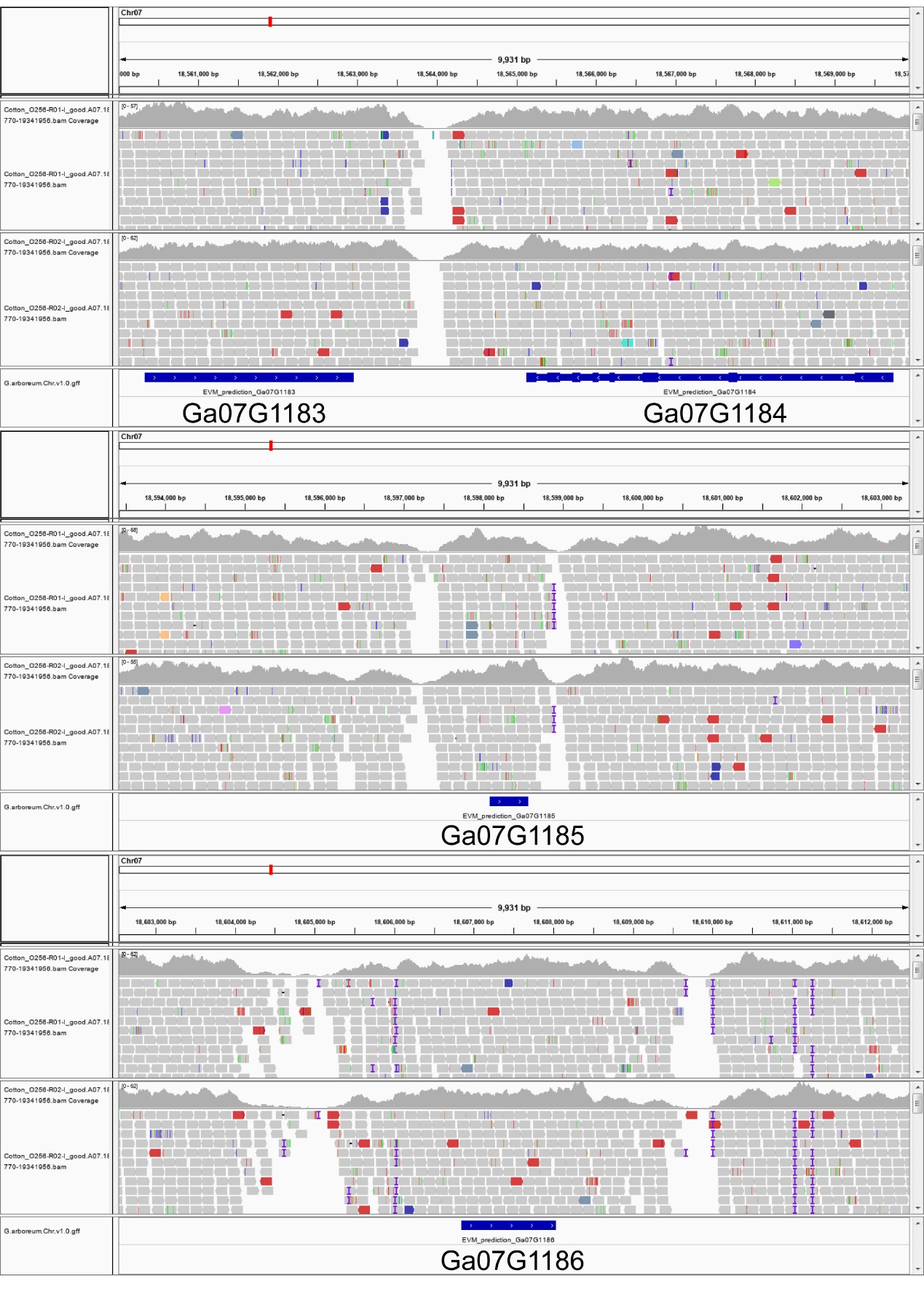

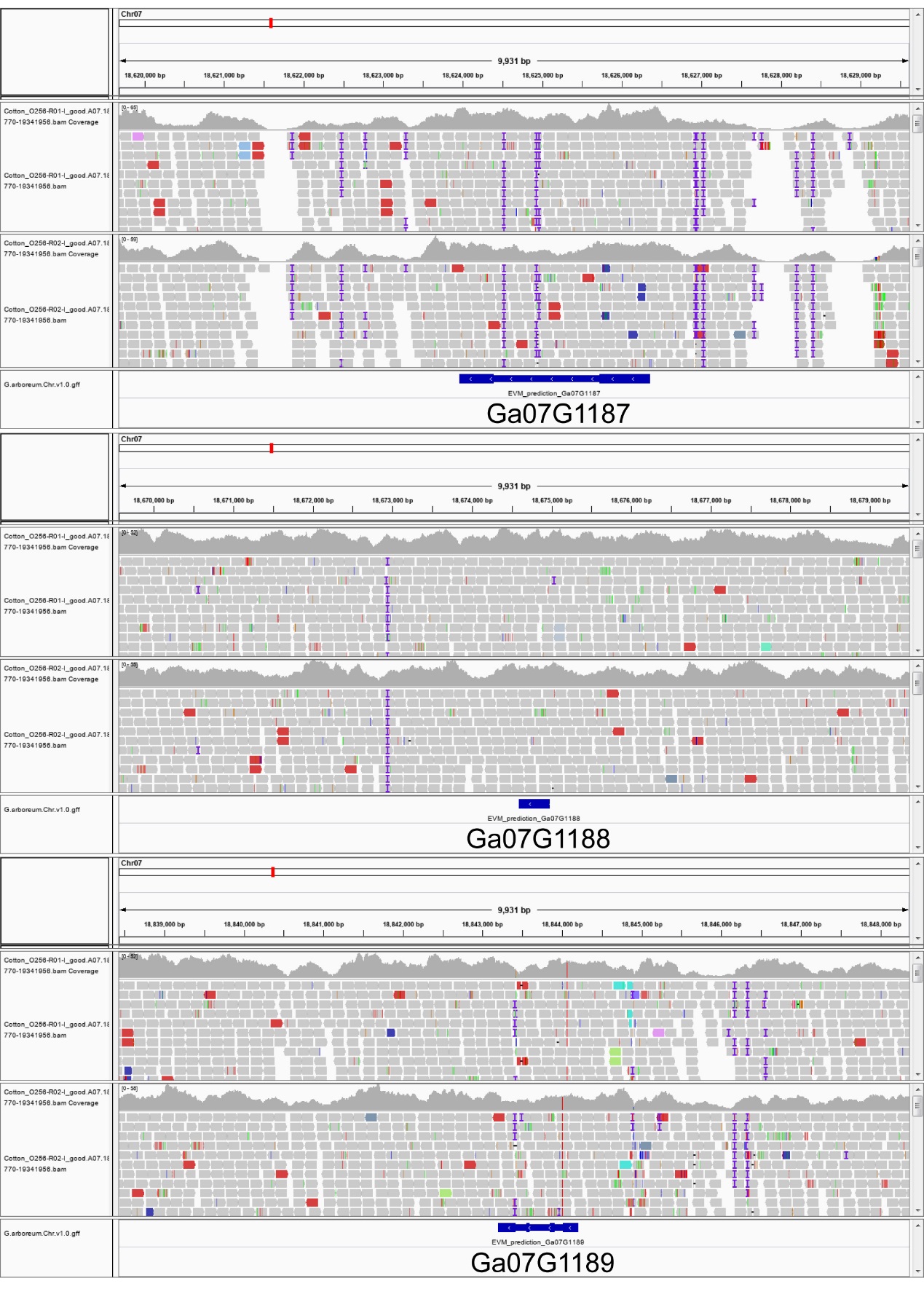

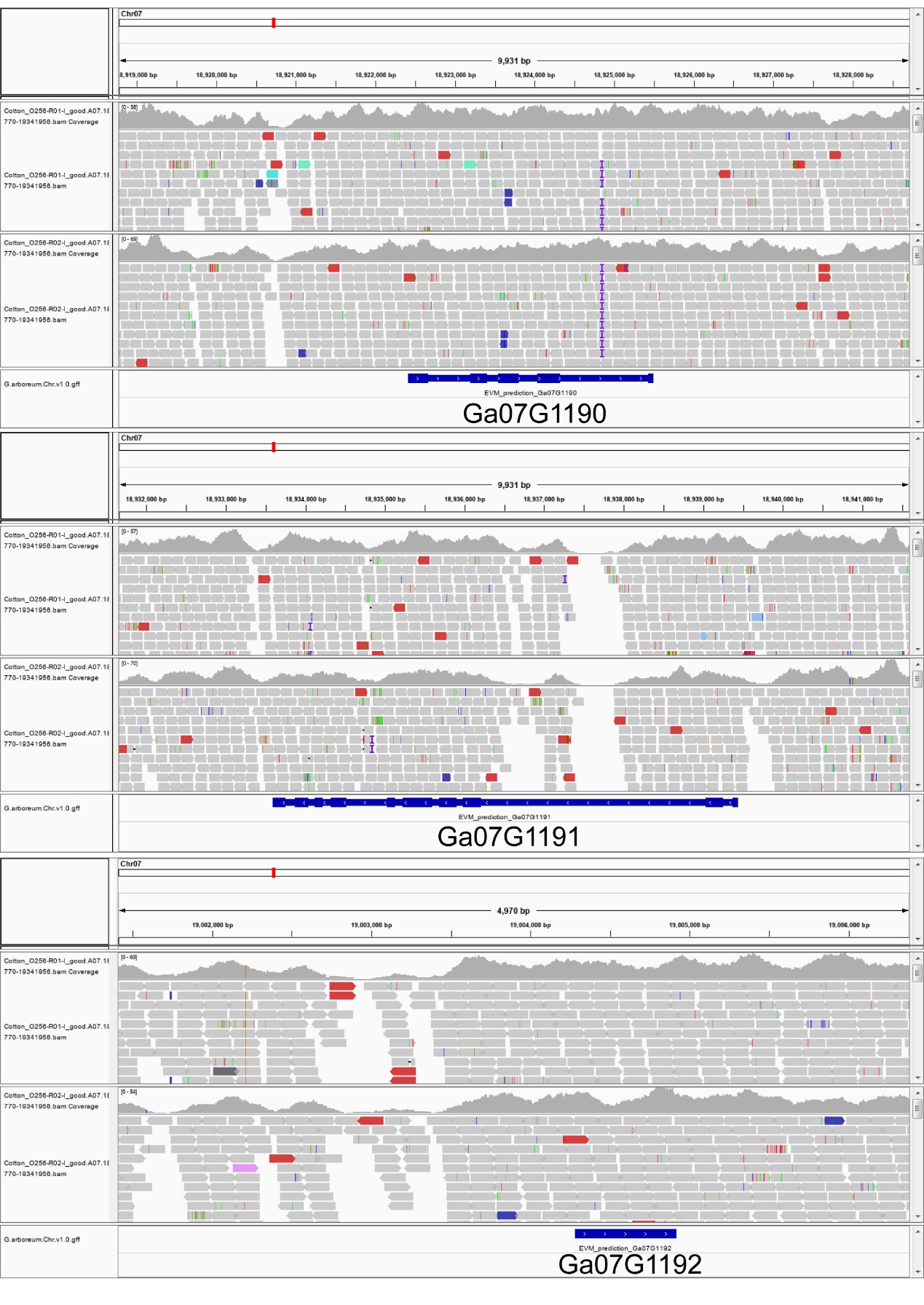

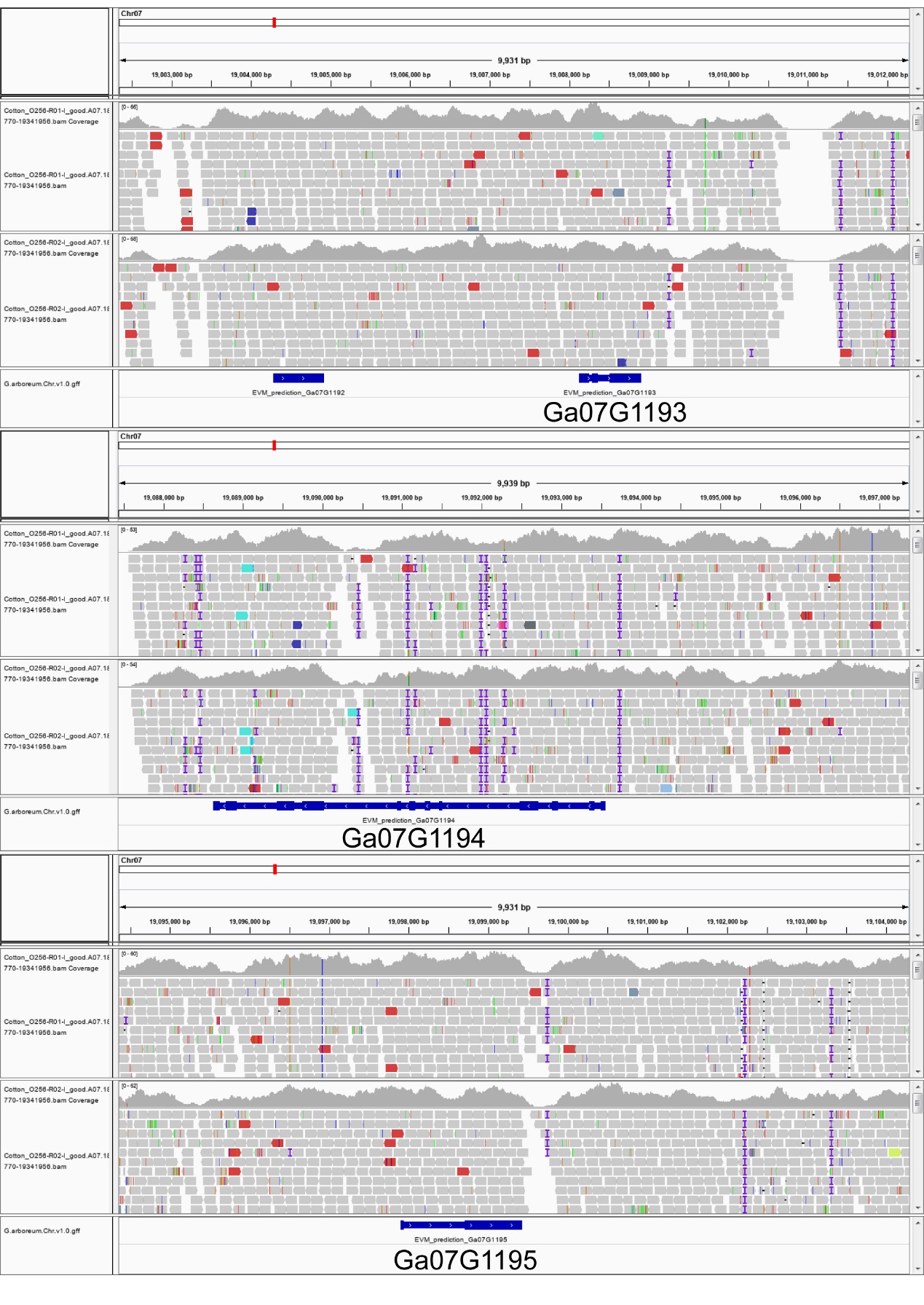

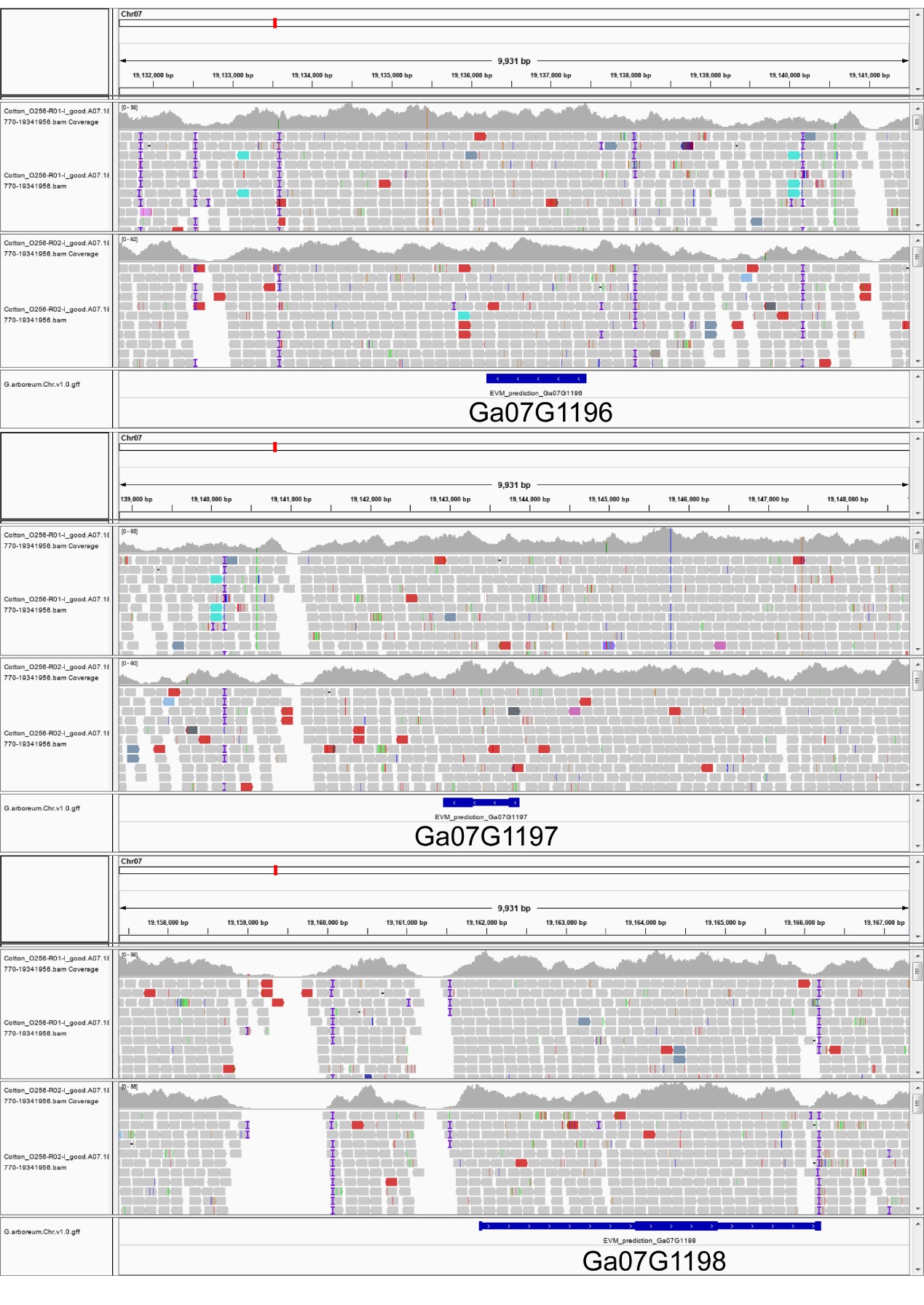

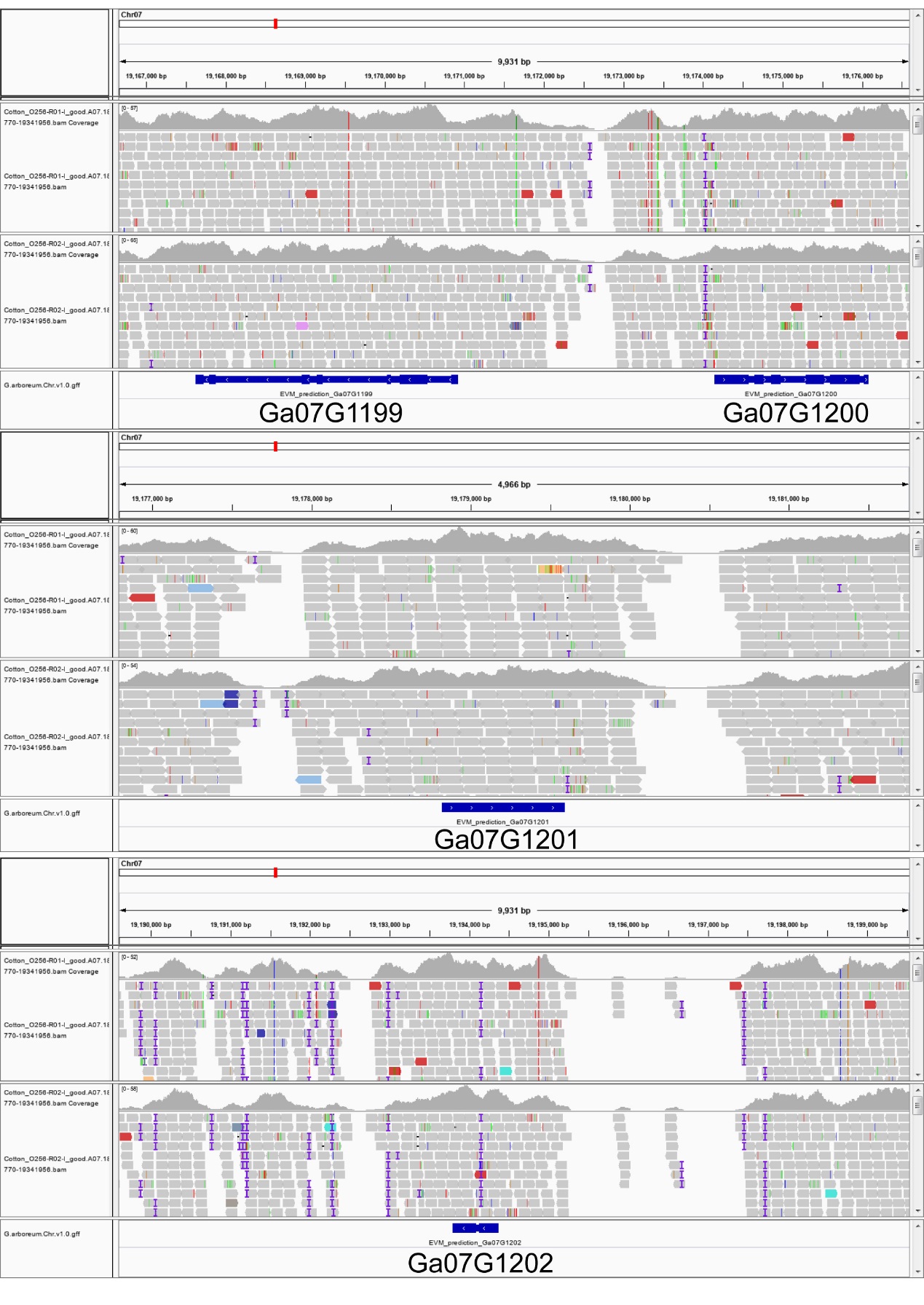

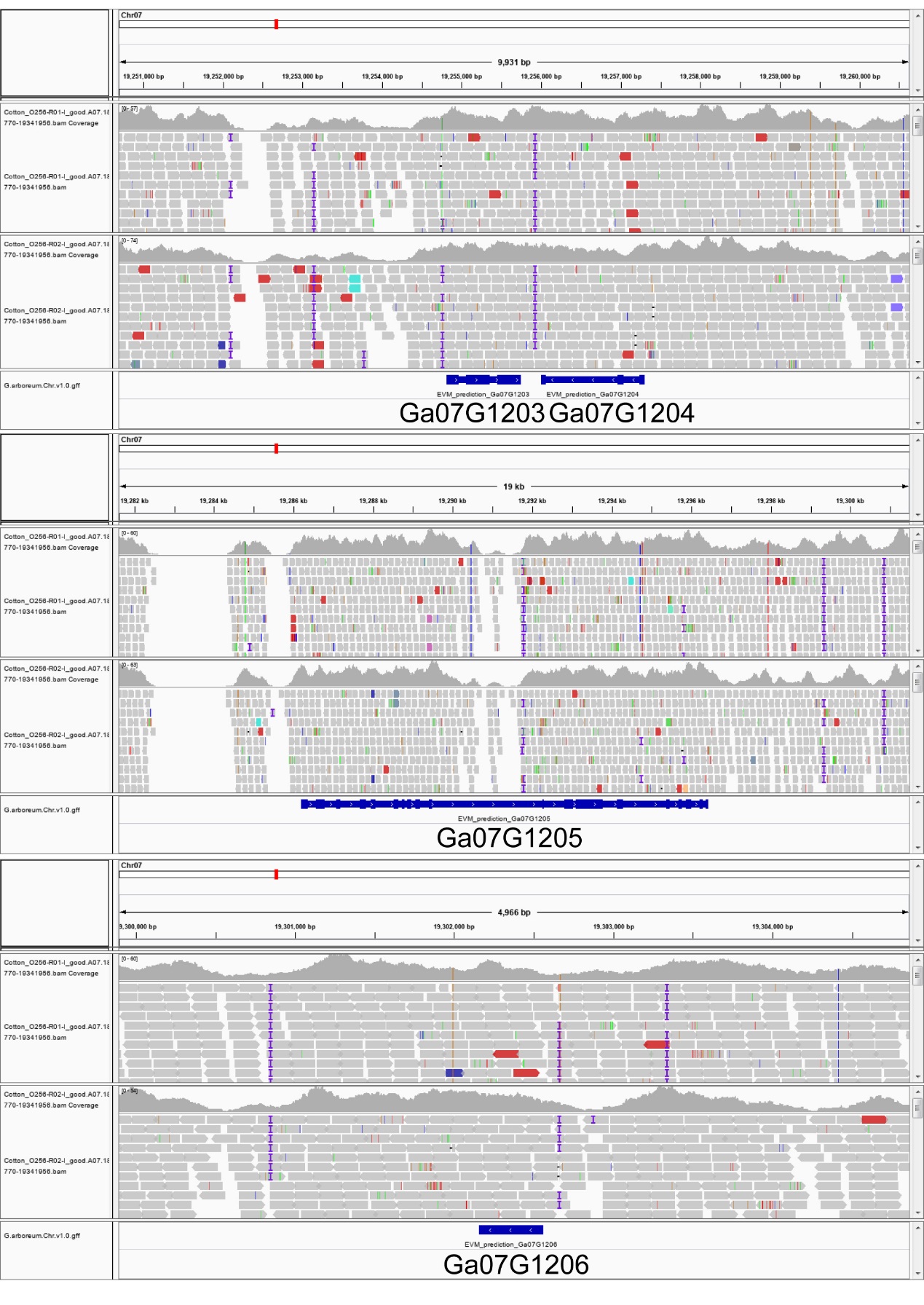

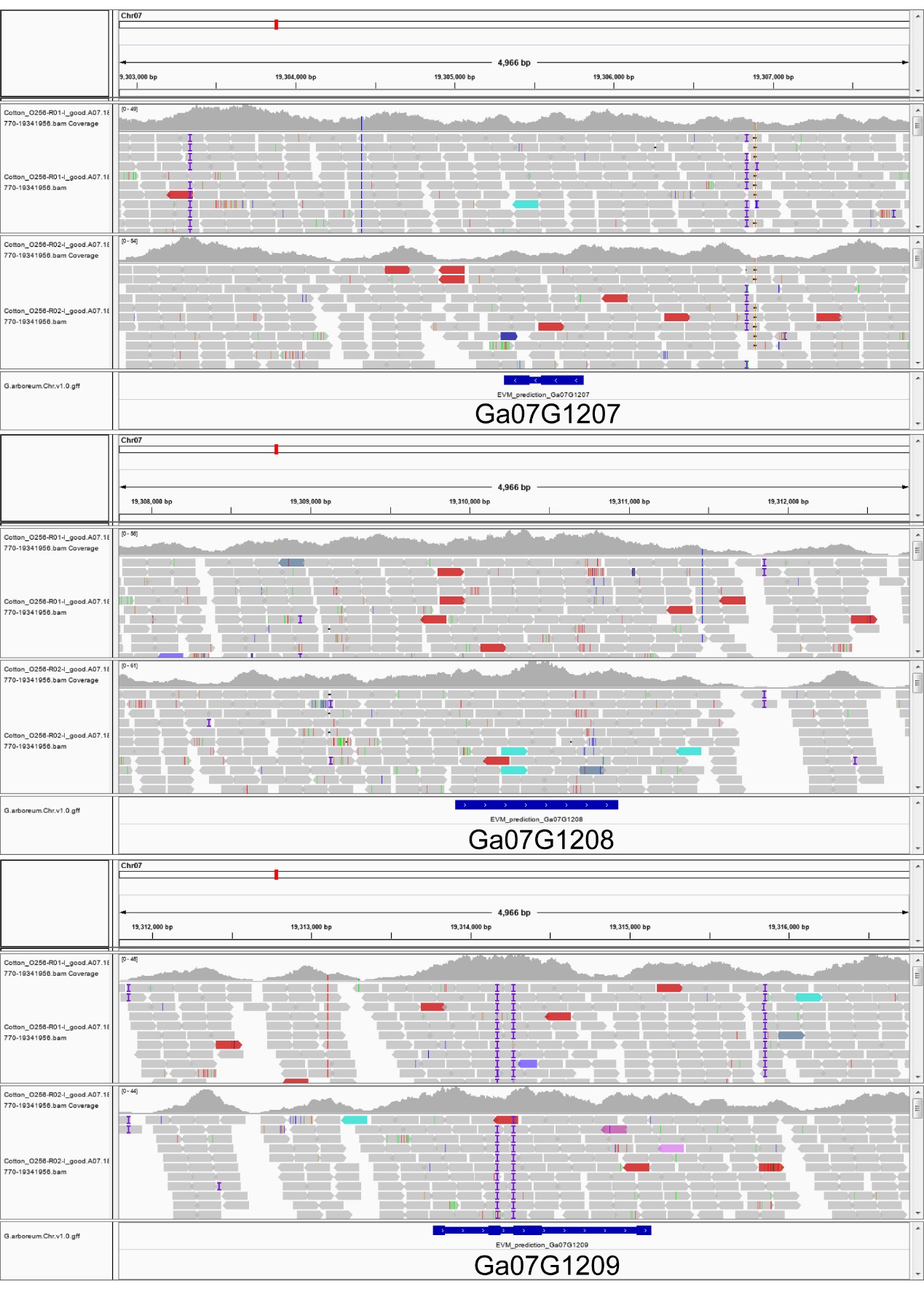

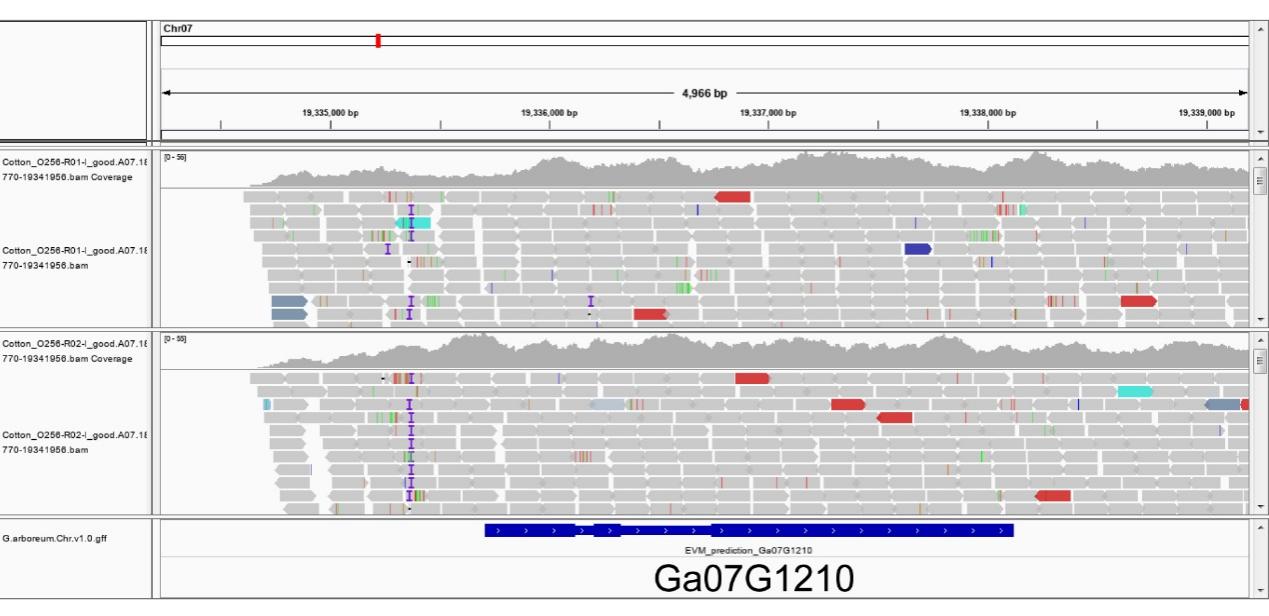


**Figure S1.** Integrative Genomics Viewer (IGV) analysis for the resequencing data of the 28 candidate genes. The SNP sites between the parent and the reference genome were marked by red lines.


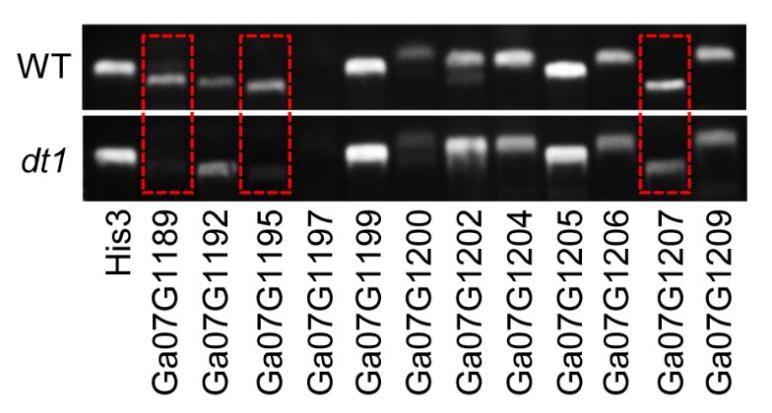


**Figure S2.** Semi-quantitative RT-PCR analysis of the candidate genes between the *dt1* mutant and the wild-type Shixiya 1. The red dot box suggests the difference between the *dt1* and the WT.

**(a)**

**(b)**

**Figure S3.** Sequence alignment of *Ga07G189* between the *dt1* mutant and wild type Shixiya 1. **(a)** Coding sequence (CDS) alignment of *Ga07G189* between the *dt1* mutant and Shixiya 1, a 93-bp deletion was observed in the *dt1* mutant, **(b)** Full genomic sequence alignment of *Ga07G1189* between the *dt1* mutant and Shixiya 1, a SNP (G to A) was observed in the *dt1* mutant. The CDS sequence and the genome sequence of *Ga07G1189* were obtained by RT-PCR and genome PCR, and used here, respectively.


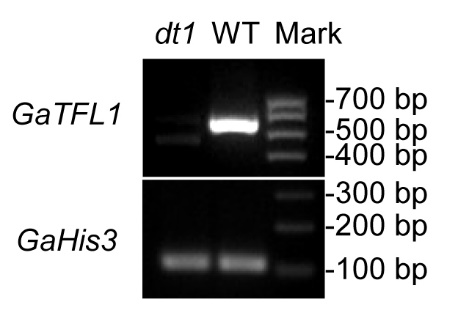


**Figure S4.** Semi-quantitative RT-PCR analysis of the *GaTFL1* between the *dt1* mutant and the wild-type Shixiya 1.
